# In vivo self-assembly and delivery of VEGFR2 siRNA-encapsulated small extracellular vesicles for lung metastatic osteosarcoma therapy

**DOI:** 10.1038/s41419-023-06159-3

**Published:** 2023-09-22

**Authors:** Lingfeng Yu, Gentao Fan, Qingyan Wang, Yan Zhu, Hao Zhu, Jiang Chang, Zhen Wang, Shoubin Zhan, Xianming Hua, Diankun She, Jianhao Huang, Yicun Wang, Jianning Zhao, Chen-Yu Zhang, Xi Chen, Guangxin Zhou

**Affiliations:** 1 Department of Orthopedics, Jinling Hospital, Affiliated Hospital of Medical School, Nanjing University, Nanjing, Jiangsu 210002 China; 2 Nanjing Drum Tower Hospital Center of Molecular Diagnostic and Therapy, Jiangsu Engineering Research Center for MicroRNA Biology and Biotechnology, School of Life Sciences, Nanjing University, Nanjing, Jiangsu 210023 China; 3 Hepatobiliary Center, The First Affiliated Hospital of Nanjing Medical University; Key Laboratory of Liver Transplantation, Chinese Academy of Medical Sciences, Nanjing, Jiangsu 210029 China; 4 State Key Laboratory of Pharmaceutical Biotechnology, Nanjing University, Nanjing, Jiangsu 210023 China; 5 Wuxi Xishan NJU Institute of Applied Biotechnology, Wuxi, Jiangsu 214101 China

**Keywords:** RNAi therapy, Tissue engineering

## Abstract

The prognosis of lung metastatic osteosarcoma (OS) remains disappointing. siRNA-based gene silencing of VEGFR2 is a promising treatment strategy for lung metastatic OS, but there is a lack of safe and efficient delivery systems to encapsulate siRNAs for in vivo administration. This study presented a synthetic biological strategy that remolds the host liver with synthesized genetic circuits for efficient in vivo VEGFR2 siRNA delivery. After being taken-up by hepatocytes, the genetic circuit (in the form of a DNA plasmid) reprogrammed the liver to drive the autonomous intrahepatic assembly and encapsulation of VEGFR2 siRNAs into secretory small extracellular vesicles (sEVs), thus allowing for the transport of self-assembled VEGFR2 siRNAs towards the lung. The results showed that our strategy was superior to the positive medicine (Apatinib) for OS lung metastasis in terms of therapeutic efficacy and toxic adverse effects and may provide a feasible and viable therapeutic solution for lung metastatic OS.

## Introduction

Osteosarcoma (OS) is the most common primary malignant tumor of bone, and is characterized by a high rate of metastasis at the time of diagnosis. Early haematogenous lung metastasis is most commonly observed in OS patients, comprising more than 85% of distant metastases [1]. Conventional curative strategies for OS patients include a period of preoperative (neoadjuvant) chemotherapy, followed by complete surgical resection and postoperative (adjuvant) chemotherapy. Under the appropriate standard treatment, the five-year survival rate has stabilized between 60% and 70% in patients with localized OS but is only 20% in metastatic OS patients [2, 3]. By virtue of the current surgical techniques, it is almost impossible to remove all metastatic lesions, and drugs for chemotherapy remain almost the same as those used in the early 1980s such as methotrexate, doxorubicin, cisplatin and ifosfamide. The prognosis of OS metastatic patients has not improved over the past three decades due to limited and monotonous treatments [1], which underscores an imperative need for novel treatment strategies for refractory OS lung metastasis.

In recent years, tumor-associated angiogenesis has been found to be critical for lung relapse in patients with metastatic OS. Uncontrolled vascular growth or excessive blood vessel remodeling serves as a route of metastasis for OS tumor cells [4, 5], and during this process dormant endothelial cells adjacent to the OS lesions are activated and engage in the formation of new blood vessels. Mechanistically, VEGF (vascular endothelial growth factor) and its main receptor VEGFR2 (vascular endothelial growth factor receptor 2) are key mediators in endothelial cell-mediated angiogenesis and pulmonary metastasis in OS [6, 7]. By specifically targeting the VEGF-VEGFR2 axis, some antivascular therapeutic drugs, especially tyrosine kinase inhibitors (TKIs), have been identified and approved by the US Food and Drug Administration (FDA). Among these TKIs, apatinib (Aitan®) can highly and selectively target VEGFR2 and is highly potent in restraining VEGF-mediated endothelial cell migration and proliferation [7]. Apatinib has proven safe and efficacious for OS pulmonary metastasis in clinical trials [8–10] and is consequently included in the US National Comprehensive Cancer Network (NCCN) Guidelines (www.nccn.org) as a second-line therapy for OS. However, studies on the antiangiogenic therapeutic strategies for OS lung metastasis have been restricted by long-term therapeutic effects and safety issues and are still in the infancy stage. Intractable drug resistance leads to the poor efficacy of long-term administration in OS patients, and the incidence of some adverse drug reactions remains high, including cutaneous reactions, gastrointestinal reactions, thrombosis and cardiovascular complications [7, 11]. These issues have severely compromised the drug efficacy and further hindered the large-scale application. Thus, there is an urgent need to identify new therapeutic modalities targeting angiogenesis in the treatment of OS lung metastasis.

Small interfering RNAs (siRNAs) can specifically target tumor-related genes, especially those that are considered undruggable using conventional treatments, and some pioneering clinical studies have reported encouraging results [12, 13]. Given the fragility of naked RNA molecules in the harsh in vivo environment, a safe delivery technology for local or systemic administration of siRNA has become indispensable. Numerous delivery systems have been proposed and investigated, especially to improve the targeting ability of genes that are located outside the liver and those that cannot be reached by local administration [14–16]. However, the traditional methods for siRNA delivery cannot meet the expectations, primarily due to both the inevitable toxic adverse effects or immune incompatibility and the liver-caused decreased delivery efficiency (the so-called liver first pass effect) [17]. Given these limitations induced by foreign formulations, borrowing the body’s own shipping method, including extracellular vesicles (EVs), may serve as a promising alternative. Officially, MISEV2018 [18] and recent articles [19, 20] define extracellular vesicles approximately 100 nm (30–250 nm in diameter) as “small EVs” (sEVs). sEVs secreted by endogenous cells are natural, and one of their intrinsic roles is intercellular communication, allowing cells to exchange small RNA molecules. The cellular functions of sEVs possess great prospects in siRNA delivery for therapeutic purposes. Despite these advantages in sEV-mediated therapy, highly-efficient small RNA loading and the large-scale production and isolation of sEVs are still expensive and labor-intensive. To solve these problems, it is worthwhile to develop an efficient, handy and safe delivery system for siRNA-encapsulated sEVs.

Recently, we developed a synthetic biology strategy that reprogrammed the host liver to self-assemble siRNAs into liver-secreted sEVs for in vivo siRNA delivery to target organs [21]. The theoretical basis for this strategy is the inherent ability of the liver to express exogenous genes introduced by rapid intravenous administration of a genetic circuit in the form of naked plasmid DNA [22, 23]. In this study, we constructed a genetic circuit encoding a VEGFR2 siRNA to engineer the liver to transcribe and self-assemble VEGFR2 siRNA-encapsulated sEVs and evaluated its therapeutic value in OS lung metastasis mouse models.

## Results

### VEGFR2 expression is elevated in OS lung metastasis

Previous studies have shown that aberrantly expressed VEGFR2 in metastasis is vital in endothelial cell-mediated angiogenesis and pulmonary metastasis in OS [7, 24]. To test this hypothesis, we measured the expression levels of VEGFR2 in paired primary OS specimens and lung metastatic specimens by immunohistochemistry (IHC). VEGFR2 mainly accumulated in lung metastatic specimens but not in primary OS specimens (Fig. S1A, C). Notably, VEGFR2 was mainly located in spindle-shaped endothelial cells in metastatic lesions but not in tumor cells. A consistent trend was also observed for the expression of CD31, a surface marker of neovascular endothelial cells (Fig. S1B, C). Subsequently, we expanded the OS sample size to 12 pairs to verify the differential expression of VEGFR2 at both the protein and mRNA levels. Western blotting and quantitative reverse transcription PCR (qRT-PCR) assays revealed that VEGFR2 was significantly elevated in OS lung metastatic specimens relative to primary specimens (Fig. S1D–F). These results confirmed the increased VEGFR2 expression and vigorous new blood vessel growth in OS lung metastatic specimens, which provided a theoretical basis for the self-assembly of VEGFR2 siRNA for the treatment of OS lung metastasis.

### Design and construction of the genetic circuits

**Fig. 1:**
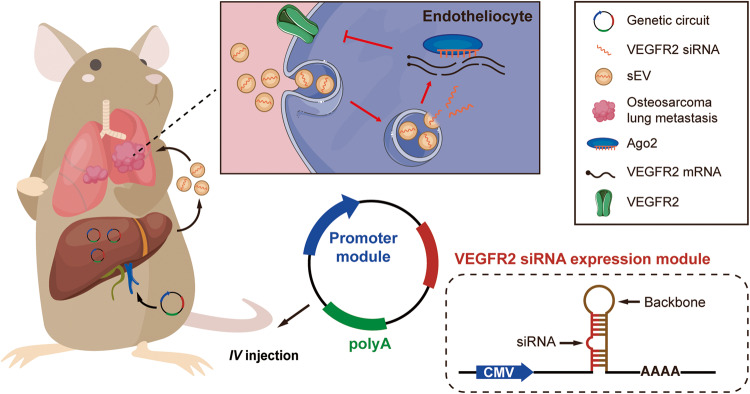
Schematic description of the architecture of the anti-VEGFR2 circuit. The genetic circuit contains two dominating functional modules: a CMV promoter and a VEGFR2 siRNA-expressing backbone. When the anti-VEGFR2 circuit is placed in the tissue classis such as the liver after intravenous administration, the CMV promoter directs the transcription of VEGFR2 siRNA and facilitates the loading of VEGFR2 siRNA into sEVs. After being secreted into the circulation, VEGFR2 siRNA enclosed in sEVs accumulates in OS lung metastatic lesions, especially in vascular endothelial cells. Finally, VEGFR2 mRNA is degraded and the angiogenesis is impaired, which inhibits the lung metastasis of OS.

Our genetic circuit combined two parts: one was a siRNA-producing backbone that encoded a siRNA specifically targeting VEGFR2, and the other was a cytomegalovirus (CMV) promoter that drove the expression of VEGFR2 siRNA (Fig. 1).

In this study, we mainly selected human umbilical vein endothelial cells (HUVECs) for in vitro validation experiments and established mouse models for in vivo efficacy and toxicity verification. Therefore, we constructed a genetic circuit encoding human VEGFR2 siRNA and a genetic circuit encoding mouse VEGFR2 siRNA. First, the coding sequences of both human and mouse VEGFR2 were scanned using the DSIR (designer of siRNA) algorithm (http://biodev.cea.fr/DSIR/DSIR.html) to generate candidate VEGFR siRNAs of human origin and mouse origin, respectively, and the set of seven siRNAs with the highest scores was selected for further screening. For the siRNA-expressing structure, we embedded the candidate VEGFR2 siRNA sequences into a miR-155 precursor (pre-miR-155) backbone. Second, the CMV promoter, facilitated by an enhancer element, was employed as the driving promoter due to its well-known efficiency in transcription of the pre-miRNA backbone. By integrating these two components, a CMV-driven genetic circuit encoding different candidate VEGFR2 siRNAs was constructed (hereafter, the anti-VEGFR2 circuit). Third, the candidate genetic circuit encoding human VEGFR2 siRNA was transfected into HUVEC cells, and the candidate circuit encoding mouse VEGFR2 siRNA was transfected into murine endothelial cells (EOMA) for further screening. Notably, the genetic circuits encoding the sixth siRNA in both mouse and human shortlists showed the best knockdown efficiency (Fig. S1G–J), and these two genetic circuits encoding either mouse VEGFR2 siRNA or human VEGFR2 siRNA were applied for the following experiments.

Theoretically, when the anti-VEGFR2 circuit is ingested and processed by the liver after intravenous administration, VEGFR2 siRNA is transcribed by the CMV promoter and then packaged into sEVs. Through blood circulation, the sEVs secreted by the liver entered the inferior vena cava (IVC) and then accumulated in the lungs, especially in OS lung metastatic lesions with rich blood supply. Finally, VEGFR2 siRNA was released into tumor vessels to play a significant therapeutic role (Fig. 1).

### Genetic circuits facilitate the self-assembly and release of functional VEGFR2 siRNA in ex vivo models

**Fig. 2:**
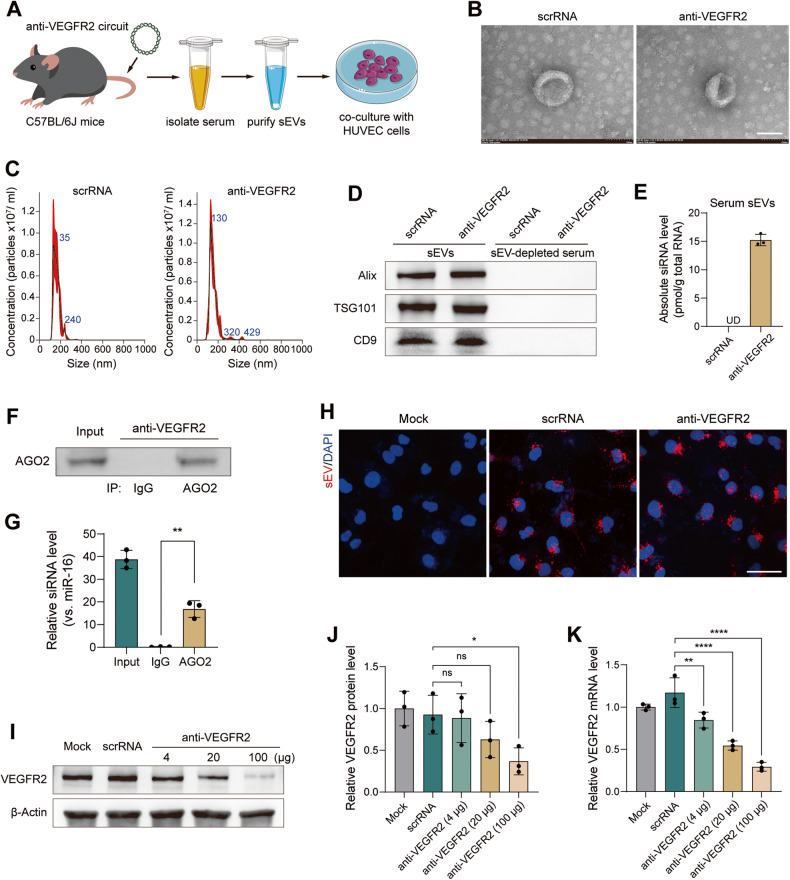
Characterization of self-assembled VEGFR2 siRNA purified from serum. **A** Schematic of the experimental design. C57BL/6 J mice were intravenously injected with scrRNA circuit or anti-VEGFR2 circuit (10 mg/kg) every 2 days for a total of seven times, and then the sEVs were purified from mouse serum and incubated with HUVECs. Next, the uptake of self-assembled VEGFR2 siRNA by HUVECs and the subsequent suppression of VEGFR2 expression by self-assembled VEGFR2 siRNA were examined in this ex vivo model. **B** Representative TEM images of serum sEVs. Scale bar: 100 nm. **C** The size distribution and concentration of serum sEVs were determined by NTA. **D** Western blot analysis of specific markers (Alix, TSG101 and CD9) in purified sEVs and sEV-depleted serum. **E** qRT-PCR analysis of VEGFR2 siRNA levels in serum sEVs (*n* = 3 per group). **F**, **G** Serum sEV RNA in C57BL/6 J mice injected with an anti-VEGFR2 circuit was subjected to immunoprecipitation using IgG or anti-AGO2 beads. Representative western blots (**F**) and qRT-PCR analysis data (**G**) are shown (*n* = 3 per group). **H** Serum sEVs were fluorescently labeled with PKH26, and PKH26-labeled sEVs were incubated with HUVECs for 6 h. The level of intracellular fluorescence intensity was monitored by confocal microscopy. Scale bar: 50 μm. **I**, **J** Western blot analysis of VEGFR2 protein levels in HUVECs after 36- h of incubation with serum sEVs. Different doses of sEVs were added to reveal the dose-dependent effect. Representative western blots (**I**) and densitometric analysis data (**J**) are shown (*n* = 3 per group). **K** qRT–PCR analysis of VEGFR2 mRNA levels in HUVECs after 36- h of incubation with different doses of serum sEVs (*n* = 3 per group). Values are presented as the mean ± SD. Significance was determined using a two-sided *t*-test in **G** or using one-way ANOVA in **J** and **K**. *P < 0.05; **P < 0.01; ****P < 0.0001; ns = not significant.

To investigate whether the in vitro-synthesized genetic circuits function well in the intricate in vivo environment, an ex vivo model was established. In this model, the anti-VEGFR2 circuit was intravenously (IV) injected into C57BL/6 J mice every two days for a total of seven times over the course of two weeks. Afterwards, the treated mice were sacrificed and circulating sEVs were extracted from the mouse serum (Fig. 2A). A CMV-directed genetic circuit encoding a scrambled RNA (scrRNA) was used as a negative control. First, the proper enrichment of serum sEVs using ultracentrifugation was confirmed. Transmission electron microscope (TEM) revealed that sEVs from both the anti-VEGFR2 circuit and scrRNA circuit groups were morphologically cup-shaped and 50–150 nm in diameter (Fig. 2B). Nanoparticle tracking analysis (NTA) demonstrated the characteristic size distribution of sEVs in each group, with a peak at ~130 nm (Fig. 2C). Western blotting revealed the presence of sEV markers (Alix, TSG101 and CD9) in the extracted sEVs but not in the sEV-depleted serum (Fig. 2D). These data demonstrated that the structure, size and biological properties of the serum sEVs were unaffected after administration of the genetic circuits. Second, we explored whether the spontaneous loading of siRNAs into sEVs could be efficiently driven by genetic circuit administration. Notably, a significant amount of VEGFR2 siRNA was detected in serum sEVs from the mice injected with the anti-VEGFR2 circuit but not in serum sEVs from the mice injected with the scrRNA circuit (Fig. 2E). Since siRNA processing relies on Argonaute 2 (AGO2) and the proper loading of siRNA onto AGO2 is expected to enhance the on-target effects of siRNA [25], an immunoprecipitation assay was performed to evaluate the interactions between endogenous AGO2 and VEGFR2 siRNA in serum sEVs. VEGFR2 siRNA was readily detected in the serum sEVs precipitated with anti-AGO2 beads in the anti-VEGFR2 circuit group (Fig. 2F, G), suggesting that our genetic circuit design guaranteed abundant loading of siRNAs in serum sEVs. Third, the purified sEVs labeled with PKH26 were incubated with HUVECs, and the intracellular fluorescence signal was detected by confocal microscopy. The serum sEVs derived from both the anti-VEGFR2 circuit and scrRNA circuit groups were similarly taken up HUVECs (Fig. 2H), indicating that incorporation of different siRNA cargoes in sEVs did not impact uptake by recipient cells. Fourth, we explored whether VEGFR2 could be knocked down by VEGFR2 siRNA-encapsulated sEVs. After incubation with serum sEVs from mice injected with the anti-VEGFR2 circuit, the protein and mRNA levels of VEGFR2 in HUVECs were remarkably reduced (Fig. 2I–K). These results reveal that the genetic circuits could drive the self-assembly of VEGFR2 siRNAs in the form of sEVs, which could be further absorbed by recipient cells to suppress VEGFR2 expression.

The liver is made up of multiple cell types, among which hepatocytes are the most abundant cell type and the most important functional unit of the liver [26]. Hence, we assumed that hepatocytes in the host liver may play a major role in the self-assembly of sEV-encapsulating VEGFR2 siRNA after genetic circuit administration. To verify this hypothesis, C57BL/6 J mice were IV injected with anti-VEGFR2 circuit or scrRNA circuit every two days for a total of seven times, and then the primary hepatocytes were extracted and cultured in vitro. The sEVs were further purified from the culture medium of primary hepatocytes and characterized (Fig. S2A). First, the primary hepatocytes extracted from mice were examined by immunofluorescence staining for albumin (ALB), a liver specific marker. Primary hepatocytes exhibited a typical cuboid or polygonal morphology, a large proportion of primary hepatocytes had a characteristic binucleate appearance, and ALB was highly expressed in these cells (Fig. S2B). Second, the size and morphology of the sEVs extracted from primary hepatocyte culture medium were assessed via TEM and NTA and sEV markers were detected by western blotting. sEVs secreted by hepatocytes from the anti-VEGFR2 circuit-treated mice displayed a morphology, size distribution and physical properties similar to those of sEVs derived from the hepatocytes of scrRNA circuit-treated mice (Fig. S2C–E). Third, qRT-PCR assays revealed that a significant amount of VEGFR2 siRNA accumulated in primary hepatocyte-derived sEVs from the anti-VEGFR2 circuit-treated mice but not in those from the scrRNA circuit-treated mice (Fig. S2F). Fourth, a routine sEV coincubation assay demonstrated that sEVs secreted by primary hepatocytes from mice injected IV with an anti-VEGFR2 circuit could be internalized by HUVECs (Fig. S2G), which subsequently diminished VEGFR2 expression (Fig. S2H–J). In conclusion, these results indicate that the anti-VEGFR2 circuit efficiently directed the self-assembly of VEGFR2 siRNAs into sEVs and that VEGFR2 siRNA transcription and assembly of VEGFR2 siRNA-encapsulated sEVs might occur in hepatocytes.

### Tracking of the delivery of self-assembled VEGFR2 siRNA in vivo

**Fig. 3:**
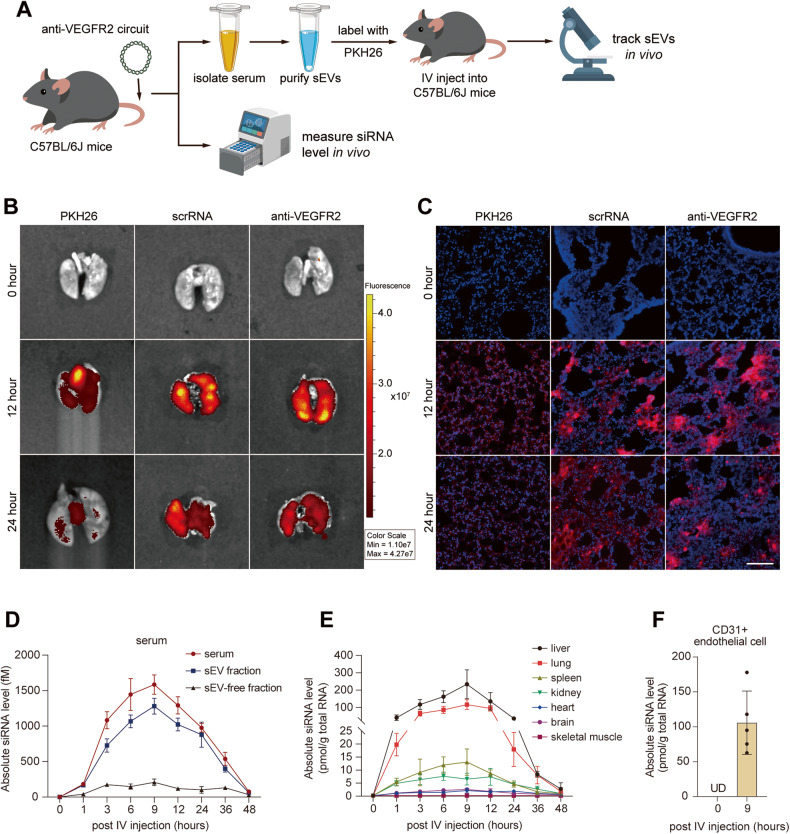
Tracking and visualization of the delivery of self-assembled VEGFR2 siRNA to the lung. **A** Experimental scheme for sEV tracking in vivo. For sEVs tracking, serum sEVs were harvested from C57BL/6 J mice injected with scrRNA circuit or anti-VEGFR2 circuit (10 mg/kg) every two days for a total of three times, fluorescently labeled with PKH26, and intravenously injected into a set of new C57BL/6 J mice. The distribution of fluorescent sEVs was determined at different time points. For VEGFR2 siRNA kinetics detection, C57BL/6 J mice were intravenously injected with an anti-VEGFR2 circuit (10 mg/kg) every two days for a total of seven times, and qRT–PCR was conducted to detect VEGFR2 siRNA levels at continuous time points. **B**, **C** In situ detection of the sEV distribution in lungs at 0, 12 and 24 h after injection of PKH26-labeled sEVs. Representative in vivo bioluminescence imaging (IVIS) (**B**) and immunofluorescence images (**C**) of PKH26-labeled sEVs (red); DAPI-stained nuclei (blue). Scale bar: 100 μm. Kinetics of VEGFR2 siRNA in the serum (**D**) and various organs (**E**) after intravenous injection of the anti-VEGFR2 circuit (10 mg/kg) into C57BL/6 J mice (*n* = 3 per group). **F** VEGFR2 siRNA level in CD31+ endothelial cells before injection, 9 h after intravenous injection of the anti-VEGFR2 circuit (10 mg/kg) into C57BL/6 J mice (*n* = 5 per group).

Considering that high enrichment of VEGFR2 siRNA in the lung is essential for the treatment of lung metastatic OS, we examined whether VEGFR2 siRNA was transported to the lung by circulating sEVs. We first visualized the in vivo distribution of sEVs. After being injected IV with anti-VEGFR2 circuit or scrRNA circuit, sEVs were extracted from C57BL/6 J mouse serum and tagged with PKH26 red fluorescent dye, followed by injecting IV into another batch of C57BL/6 J mice (Fig. 3A). Injection solely with PKH26 dye served as a control. The serum sEVs purified from both anti-VEGFR2 circuit- and scrRNA circuit-injected mice generated apparent fluorescent signals in the lung, and these signals were much stronger than those in the dye-injected group (Fig. 3B, C), indicating that circulating sEVs could be significantly enriched in the lung and that different RNA cargos in sEVs did not affect the in vivo distribution of sEVs. Subsequently, we investigated the biodistribution and dynamics of VEGFR2 siRNAs in mouse peripheral blood and various tissues during anti-VEGFR2 circuit treatment. After determining the concentration of VEGFR2 siRNA in the whole serum, serum sEV fraction and sEV-depleted serum, we showed that the concentration of VEGFR2 siRNA changed in a time-dependent manner in C57BL/6 J mice injected with anti-VEGFR2 circuit, peaking at approximately 9 h and dropping down to the background level in approximately 48 h in the serum and sEV pellets, but this change was not observed in the sEV-depleted serum (Fig. 3D). This result indicates that self-assembled siRNAs were mainly delivered by circulating sEVs. Direct measurement of VEGFR2 siRNA in multiple tissues showed the enrichment of siRNA in the liver and lung in a time-dependent manner, while little or no enrichment was observed in the heart, brain and skeletal muscle from anti-VEGFR2 circuit-injected mice (Figs. 3E and S3A, B). Furthermore, we performed magnetic beads sorting experiment to detect the enrichment level of VEGFR2 siRNA in endothelial cells. Sorted by CD31+ magnetic beads, pulmonary microvascular endothelial cells were obtained from mouse lung tissues before circuit injection, at 9 h after injection. Significant enrichment of VEGFR2 siRNA was confirmed by qRT-PCR analysis in endothelial cells at 9 h after anti-VEGFR2 circuit injection (Fig. 3F). These findings are consistent with previous research [21] and support the findings that the liver, through spontaneous and continuous release of siRNA-encapsulated sEVs, enables the transport of functional VEGFR2 siRNA into the lung.

### Silencing VEGFR2 with self-assembled VEGFR2 siRNA attenuates angiogenesis

**Fig. 4:**
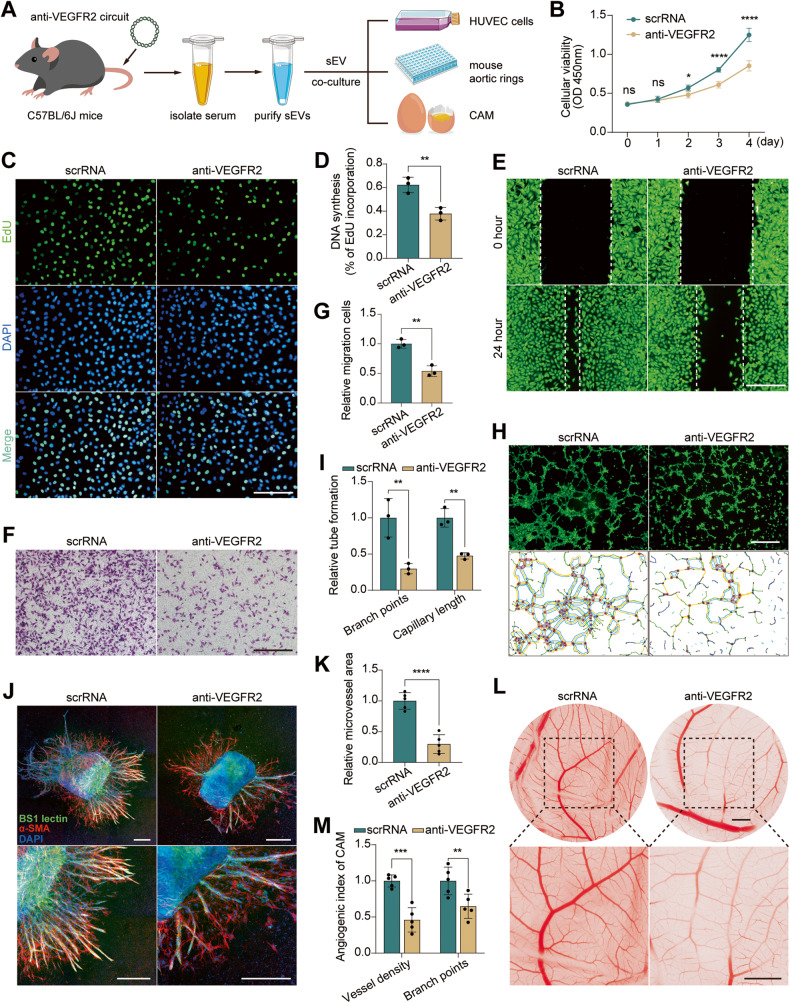
Evaluation of the antiangiogenic capacity of self-assembled VEGFR2 siRNA. **A** Schematic of the experimental design. Serum sEVs were harvested from C57BL/6 J mice injected with scrRNA circuit or anti-VEGFR2 circuit (10 mg/kg) for a total of seven times, and were subsequently subjected to evaluation of the intrinsic antiangiogenic capacity by incubating with HUVECs, mouse aortic rings and chicken allantoic membrane (CAM). **B** A CCK-8 assay was performed to estimate cell viability after incubation with serum sEVs from C57BL/6 J mice injected with scrRNA circuit or anti-VEGFR2 circuit at different time points in HUVECs (*n* = 6 per time point). **C**, **D** A EdU assay was applied to estimate cell proliferation after incubation with serum sEVs in HUVECs. S-phase entry is visualized by EdU incorporation (green); DAPI-stained nuclei (blue). Scale bar: 200 μm. Quantification of EdU incorporation is shown in **D** (*n* = 3 per group). **E**–**G** Wound healing and Transwell migration assays exhibited cell migration capability after incubation with serum sEVs in HUVECs. Scale bars: 200 μm (**E**) and 400 μm (**F**). Quantification of Transwell migration is shown in **G** (*n* = 3 per group). **H**, **I** Tube formation assay showed cell tube formation ability after incubation with serum sEVs in HUVECs. Scale bar: 1 mm. Image quantification was conducted as described in methods (**I**) (*n* = 3 per group). **J**, **K** Mouse aortic ring assay revealed the angiogenic ability of mouse aortic rings after incubation with serum sEVs. Representative immunofluorescence images (**J**). BS1 lectin-FITC (green) indicates endothelial sprouts; α-SMA represents supporting cells (red); DAPI-stained nuclei (blue). Scale bar: 400 μm. Quantification of aortic ring microvessel area as described in methods (**K**) (*n* = 5 per group). **L**, **M** The CAM assay revealed the angiogenic ability of CAM after incubation with serum sEVs. Representative images of CAM photographed on plastic dishes after resection from eggs (**L**). Scale bar: 2 cm. The statistical results of the CAM assay (**M**) (*n* = 5 per group). Values are presented as the mean ± SD. Significance was determined using a two-sided *t*-test in **B**, **D**, **G** and **K** or using one-way ANOVA in **I** and **M**. *P < 0.05; **P < 0.01; ***P < 0.001; ****P < 0.0001; ns = not significant.

Because conspicuous angiogenesis promotes OS pulmonary metastasis [5, 27, 28] and because VEGFR2 is essential for intracellular adenosine‐mediated angiogenesis and is dominantly expressed on endothelial cells [29, 30], we next examined the anti-angiogenetic potential of the anti-VEGFR2 circuit. After being purified from C57BL/6 J mice injected IV with anti-VEGFR2 circuit, a high-concentration (100 μg total protein) of VEGFR2 siRNA-encapsulated sEVs was incubated with both HUVECs and CD31+ pulmonary microvascular endothelial cells to test whether the VEGFR2 siRNA enrichment level was consistent in the two cell types (Fig. S4A). As indicated by the qRT-PCR result, there was no difference in VEGFR2 siRNA levels between two cell types (Fig. S4B). Therefore, HUVEC was selected as the cell model for the following in vitro angiogenesis experiments (Fig. 4A). First, the effect of VEGFR2 siRNA-encapsulated sEVs on the proliferation ability of HUVECs was evaluated by CCK-8 and EdU assays. The proliferation rate and DNA incorporation were significantly reduced in HUVECs incubated with sEVs from anti-VEGFR2 circuit-injected mice compared with those from scrRNA circuit-injected mice (Fig. 4B–D). Second, wound healing and Transwell migration assays revealed that the migration ability of HUVECs was diminished when incubated with sEVs from anti-VEGFR2 circuit-injected mice (Fig. 4E–G). Third, tube formation assays revealed that the capillary length and branch points of HUVECs were significantly reduced when incubated with sEVs from anti-VEGFR2 circuit-injected mice (Fig. 4H, I). Fourth, an ex vivo mouse aortic ring assay and an in vivo chick chorioallantoic membrane (CAM) assay showed that the microvessel area was substantially reduced in aortic rings incubated with sEVs from anti-VEGFR2 circuit-injected mice (Fig. 4J, K), and newly formed vessels in the CAM were also largely compromised after incubation with sEVs from anti-VEGFR2 circuit-injected mice (Fig. 4L–M). Overall, these results confirm the antiangiogenic effects of anti-VEGFR2 circuit treatment.

### Silencing VEGFR2 with self-assembled VEGFR2 siRNA inhibits OS lung metastasis in vivo

**Fig. 5:**
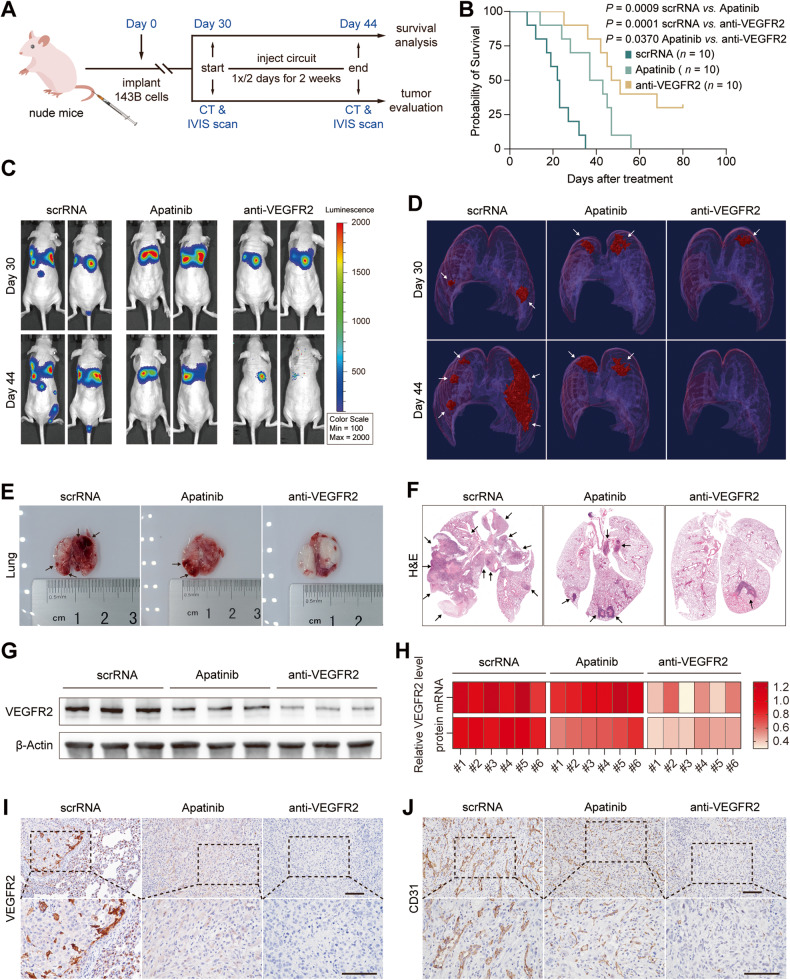
Evaluation of the therapeutic efficacy of self-assembled VEGFR2 siRNA in the tail vein OS metastasis model. **A** Flow chart of the experimental design. The OS lung metastasis model was constructed by injecting stable 143B cells labeled with firefly luciferase into the lateral tail vein of nude mice. Thirty days after injection, tumor-burdened mice were randomly divided into two groups for either survival analysis or tumor evaluation according to pulmonary tumor burden estimation by IVIS and micro-CT scans. Mice were then intravenously injected with the scrRNA circuit or anti-VEGFR2 circuit (10 mg/kg) or intragastrically administered 200 mg/kg apatinib every 2 days for a total of seven treatments. After treatment, the survival condition, tumor growth and VEGFR2 expression levels were evaluated. **B** Survival ratio of the mice treated with genetic circuits or apatinib (*n* = 10 per group). **C** Representative IVIS images of lung metastases of mice pre- (day 30) and posttreatment (day 44) with the genetic circuits or apatinib. **D** Representative 3-D reconstructions of lung metastases pre- (day 30) and posttreatment (day 44) with the genetic circuits or apatinib. White arrow shows the tumor. **E**, **F** Representative images of collected lungs and hematoxylin and eosin (H&E) staining. White dots in **E** are light reflections, black arrow shows the tumor. **G** Western blot analysis of VEGFR2 protein levels in lung metastatic samples. Representative western blots are shown. **H** Heat-map showing the quantitation of VEGFR2 mRNA levels and protein levels in lung metastatic samples (*n* = 6 per group). The detailed data quantifications are shown in Fig. S4C, D. **I**, **J** Representative images of IHC staining for VEGFR2 and CD31 protein in lung metastasis sections. Scale bar: 100 μm.

Next, we examined the therapeutic potential of the anti-VEGFR2 circuit in two mouse models of OS lung metastasis. First, we established the tail vein injection lung metastasis model (experimental lung metastasis) to mimic the latter stage of OS metastasis. The tumor burden in the lung was estimated by in vivo bioluminescence imaging (IVIS) and micro-CT scan 30 days after injection to ensure the success of lung metastasis modeling. Then, mice were injected IV with scrRNA circuit or anti-VEGFR2 circuit every 2 days for 7 times until day 44 (Fig. 5A), and the tumor-burdened mice were randomly divided into two groups for either survival analysis or tumor evaluation. Apatinib (Aitan®), a tyrosine kinase inhibitor that specifically targets VEGFR2 [7], was intragastrically administered every two days seven times as a control. The overall survival rate was modestly increased in mice treated with apatinib compared to the control, while the mice treated with the anti-VEGFR2 circuit exhibited a much longer survival time than the control, with 3 out of the 10 mice surviving for more than 80 days (Fig. 5B). Continuous body weight loss was observed over time in mice treated with scrRNA circuit, while treatment with anti-VEGFR2 circuit or apatinib significantly relieved body weight loss in tumor-bearing mice. In particular, anti-VEGFR2 circuit treatment led to moderate weight gain in some mice (Fig. S5A). Furthermore, the change in OS lung metastasis was monitored in two independent dimensions, including the luciferase intensities in IVIS assays and tumor volume in micro-CT scans. The OS metastatic lesions in apatinib-treated mice were modestly reduced in terms of the luciferase intensity (Figs. 5C and S5B) and tumor-burdened volume (Figs. 5D and S5C), whereas a remarkable decline was observed in mice treated with the anti-VEGFR2 circuit. A similar tendency of tumor shrinkage was also confirmed by histology in anti-VEGFR2 circuit-treated mice (Fig. 5E, F). At the molecular level, the mRNA and protein levels of VEGFR2 in OS metastatic lesions were evaluated. Significant downregulation of VEGFR2 mRNA and protein levels was detected in lung lesions from mice treated with the anti-VEGFR2 circuit, while only a slight reduction in VEGFR2 protein levels was observed in lung lesions from apatinib-treated mice (Figs. 5G, H and S5D, E), because apatinib is a tyrosine kinase inhibitor that mainly functions at the protein level by affecting enzyme activity. A consistent loss of VEGFR2 protein in anti-VEGFR2 circuit-treated mice was further confirmed by IHC staining (Figs. 5I and S5F). Likewise, diminished new blood vessels (CD31) (Figs. 5J and S5G) and a decreased cell proliferation rate (Ki-67) (Figure S5H, L) were observed in OS lung lesions from mice treated with the anti-VEGFR2 circuit and apatinib. Furthermore, epithelial-to-mesenchymal transition (EMT), a critical step in the invasion and metastasis of various cancers characterized by low expression of E-cadherin and high expression of the EMT-induced markers N-cadherin and vimentin, was evaluated. Reduced EMT, characterized by an increase in E-cadherin expression and loss of N-cadherin and vimentin expression, was detected in OS lung lesions from mice treated with the anti-VEGFR2 circuit and apatinib (Fig. S5I–K, M–O).

**Fig. 6:**
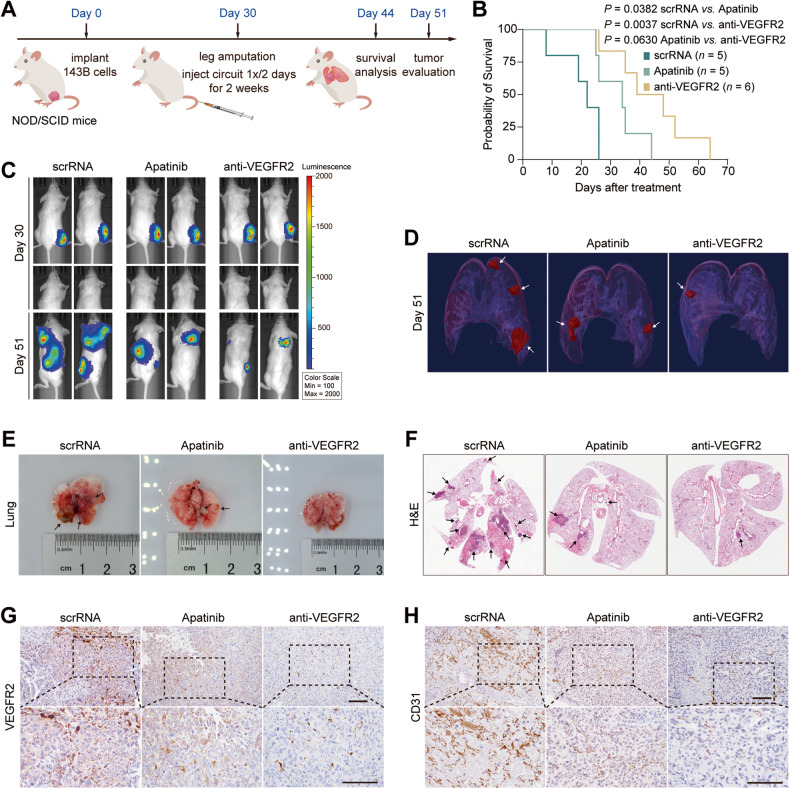
Evaluation of the therapeutic efficacy of self-assembled VEGFR2 siRNA in the spontaneous OS metastasis model. **A** Flow chart of the experimental design. The OS orthotopic xenograft tumor model was constructed by injecting stable 143B cells labeled with firefly luciferase into the tibial bone marrow cavity of NOD/SCID mice. Thirty days after injection, the tumor-bearing right legs of all mice were amputated aseptically. All mice were randomly divided into two groups for either survival analysis or tumor evaluation according to pulmonary tumor burden. Mice were then intravenously injected with the scrRNA circuit or anti-VEGFR2 circuit (10 mg/kg) or intragastrically administered 200 mg/kg apatinib every 2 days for a total of seven treatments. After treatment, the survival condition, tumor growth and VEGFR2 expression levels were evaluated. **B** Survival ratio of the mice treated with genetic circuits or apatinib (*n* = 5 in scrRNA and apatinib group, *n* = 6 in anti-VEGFR2 group). **C** Representative IVIS images of lung metastases of mice pre- (day 30) and posttreatment (day 51) with the genetic circuits or apatinib. **D** Representative 3-D reconstructions of lung metastases posttreatment with the genetic circuits or apatinib on day 51. White arrow shows the tumor. **E**, **F** Representative images of collected lungs and H&E staining. White dots in **E** are light reflections, black arrow shows the tumor. **G**, **H** Representative images of IHC staining for VEGFR2 and CD31 protein in lung metastasis sections. Scale bar: 100 μm.

Second, the therapeutic efficacy of anti-VEGFR2 circuit was evaluated in spontaneous OS lung metastasis model. We treated mice after amputation with scrRNA circuit, anti-VEGFR2 circuit or apatinib 7 times over 2 weeks, and randomly divided all the mice into two groups for either survival analysis or tumor evaluation (Fig. 6A). Compared to the mice treated with scrRNA circuit or apatinib, mice treated with the anti-VEGFR2 circuit exhibited a markedly prolonged over survival rate (Fig. 6B). After seven rounds of treatment, the proportion of mice with detectable metastasis to lung was assessed by IVIS imaging. 13 out of the 15 mice (87%) developed lung metastasis after scrRNA circuit treatment, 8 out of the 15 mice (53%) developed lung metastasis after apatinib treatment and 6 out of the 15 mice (40%) developed lung metastasis after anti-VEGFR2 circuit treatment, indicated that anti-VEGFR2 circuit treatment significant reduced spontaneous lung metastasis. Consistently, sustained weight loss was observed over time in tumor-bearing mice treated with the scrRNA circuit. In contrast, treatment with apatinib slightly relieved the weight loss, while treatment with anti-VEGFR2 circuit significantly developed weight gain (Fig. S6A). As manifested by the IVIS assay and micro-CT scans, more extensive lung metastasis and larger tumor mass were observed in mice treated with the scrRNA circuit and apatinib, while lung metastasis was dramatically limited and tumor size were significantly reduced by the treatment with the anti-VEGFR2 circuit (Figs. 6C, D and S6B, C). Furthermore, histopathological analysis also revealed fewer and smaller metastatic foci in mice treated with the anti-VEGFR2 circuit versus the mice treated with the scrRNA circuit or apatinib (Fig. 6E, F). At the molecular level, a significant decrease in VEGFR2 expression and concomitant decrease in CD31 and Ki67 levels were observed in the anti-VEGFR2 circuit-treated mice (Figs. 6G, H and S6D–G). In conclusion, all these findings demonstrate that the therapeutic effect of the self-assembled VEGFR2 siRNA is superior to that of apatinib, which may serve as a novel therapeutic tool for OS lung metastasis.

### VEGFR2 siRNA-encapsulated sEVs are self-assembled in a nontoxic and safe manner

**Fig. 7:**
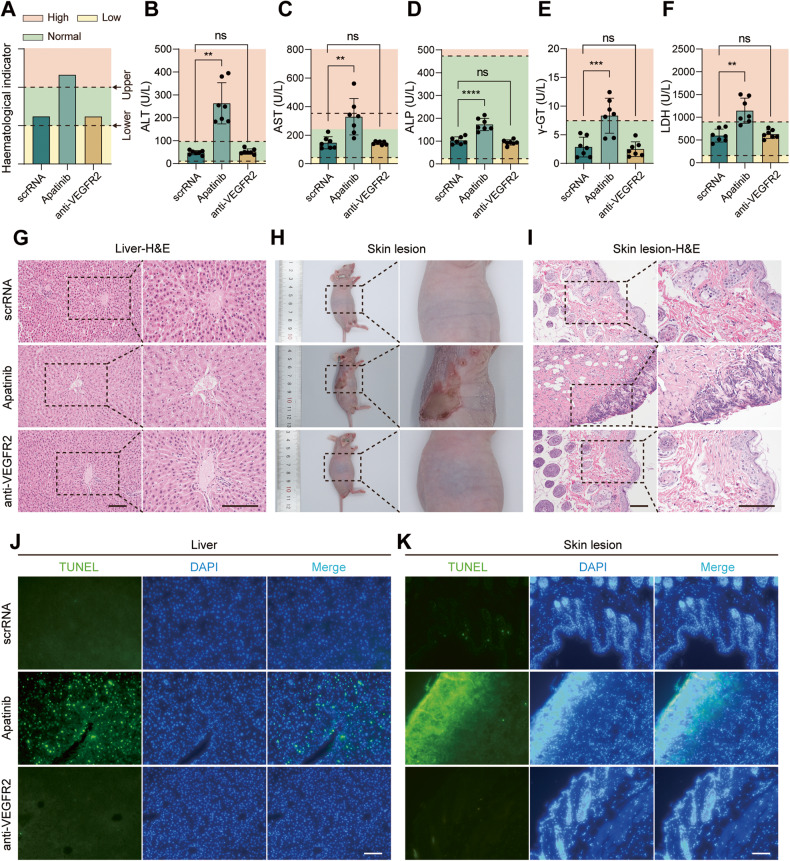
Evaluation of the toxic effects and tissue damage of self-assembled VEGFR2 siRNA in vivo. C57BL/6 J mice were intravenously injected with scrRNA circuit or anti-VEGFR2 circuit (10 mg/kg) or intragastrically administered 200 mg/kg apatinib every 2 days for a total of seven times. After treatment, mice were sacrificed, and blood and tissue samples were collected and analyzed for serum biochemical indicators and tissue damage. **A** Schematic diagram of expression level of biochemical markers. Red, green and yellow shaded areas represent the high level, normal level and low level respectively. Two dotted lines represent the lower and upper limits of the normal range. Biochemical indicators measured in serum include (*n* = 7 per group): **B** ALT (normal range: 10.06-96.47 U/L), **C** AST (normal range: 36.31-235.48 U/L), **D** ALP (normal range: 22.52-474.35 U/L), **E** γ-GT (normal range <7.78 U/L) and **F** LDH (normal range: 157.41-899.72 U/L). **G** Histological examination of the livers from genetic circuits or apatinib-treated mice. Scale bar: 100 μm. Gross specimens (**H**) and histological examination (**I**) of the skin reactivity from genetic circuits or apatinib-treated mice. Scale bar: 100 μm. **J**, **K** Representative images of TUNEL stained images of the livers (**J**) and skin tissues (**C**) from genetic circuits or apatinib-treated mice. Scale bar: 100 μm. Values are presented as the mean ± SD. Significance was determined using one-way ANOVA in **B**, **C**, **D**, **E** and **F**. **P < 0.01; ***P < 0.001; ****P < 0.0001; ns = not significant.

We next examined whether the genetic circuits can generate self-assembled VEGFR2 siRNA in a biocompatible and safe manner. First, C57BL/6 J mice were injected IV with scrRNA circuit or anti-VEGFR2 circuit or intragastrically administreted apatinib every two days for a total of seven times, and then peripheral blood and multiple organ tissues were collected for comprehensive evaluation. Representative serum biochemical markers for liver function, including alanine transaminase (ALT), aspartate aminotransferase (AST), alkaline phosphatase (ALP), gamma-glutamyltransferase (γ-GT), total bilirubin (TBIL) and direct bilirubin (DBIL); biochemical markers for kidney damage, including serum creatinine (CREA) and blood urea nitrogen (BUN); and cardiac injury markers, including creatine kinase (CK) and lactate dehydrogenase (LDH), were examined. Treatment with apatinib caused a remarkable increase in liver injury markers (ALT, AST, ALP and γ-GT in particular) and the cardiac injury marker LDH, whereas treatment with the anti-VEGFR2 circuit did not result in significant hepatic, renal or cardiac toxicities (Figs. 7A–F and S7A–E). No abnormal hematologic results were observed in routine blood analysis for either the anti-VEGFR2 circuit or apatinib (Fig. S7F–H). Second, histological examination was conducted to check for tissue damage in vital organs (heart, liver, lung and kidney). Notably, modest hepatocyte damage was observed in the livers of mice treated with apatinib, as represented by the loss of the normal hepatic cord or plate, blurred cell membrane boundaries, hepatocellular vacuoles and nuclear condensation. In contrast, continuous treatment with the anti-VEGFR2 circuit did not cause noticeable tissue damage (Figs. 7G and S7I). Moreover, the apatinib therapeutic regimen caused severe skin reactions in multiple locations (chest, upper back and upper extremities) (Figs. 7H and S7J). Representative pathologic examination showed grievous destruction in the skin structure of the upper back, in which the missing epidermis was superseded by ulcerated or necrotic tissue; hyperplasia and structural disorder were observed in the collagen and fibrous tissue of the superficial dermis; the number of skin appendages was decreased or even disappeared; and a large number of chronic inflammatory cells and histocytes (fibroblasts, myofibroblasts) aggregated and proliferated in necrotic skin (Fig. 7I). In sharp contrast, the anti-VEGFR2 circuit caused no toxic adverse effects on the skin. TUNEL assay was further applied to detect cell apoptosis in multiple organs. We noted that there were a large number of apoptotic cells in the livers and skin defect sites of mice treated with apatinib (Fig. 7J, K), which was consistent with the results of pathologic examination. Besides, multiple apoptotic cells were also found in kidneys and hearts in addition to lung tissues from mice treated with apatinib (Fig. S8), while the treatment with the anti-VEGFR2 circuit did not cause significant apoptosis in any organ. Overall, these results demonstrate that the self-assembled VEGFR2 siRNA could be applied in vivo without causing undesirable tissue damage or toxic adverse effects.

## Discussion

VEGFR2 is one of the most important receptors in VEGF-induced angiogenesis which is mainly expressed in microvascular endothelial cells and endothelial progenitor cells [4, 11]. The anomalously high expression of VEGFR2 was a negative prognostic biomarker of OS and correlated with both lung metastasis and poor prognosis [24, 31–33]. Our study confirmed that endothelial cell localized-VEGFR2 was widely expressed in lung metastases compared to local OS samples. Therefore, our in vivo self-assembled siRNA strategy is to target VEGFR2 in endothelial cells and achieved good therapeutic effect. But We have to admit that the specific localization and mechanism of VEGFR2 in various cell subsets of OS still remains understudied. One the one hand, VEGFR2 is generally considered to be expressed on the surface of bone marrow-derived (BMD) endothelial progenitor cells and mature endothelial cells, which initiate the premetastatic OS niche and further facilitate the vascularization of metastatic lesions [33–35]. Recent single-cell RNA sequencing analysis of OS specimen also confirmed the aberrant expression of VEGFR2 in endothelial-like cells [36]. On the other hand, Guo et al [24, 31, 32] reported that VEGFR2 could also be localized in OS cells. Specifically, VEGFR2 was highly expressed in some OS cell lines (MG-63, KHOS and U-2 OS) and the anomalously high expression of VEGFR2 was a negative prognostic biomarker of OS and correlated with both lung metastasis and poor prognosis. Therefore, both expression pattern and molecular mechanism of VEGFR2 in the OS microenvironment remain to be elucidated.

The liver was chosen as the synthesis arsenal to direct the formation of VEGFR2 siRNA-encapsulated sEVs after taking up the genetic circuits in the form of plasmid DNA. Because the liver can express transgenes introduced by intravenous administration of plasmid DNA, and high level of gene expression in hepatocytes are achieved by rapid tail vein injection of large volume of plasmid DNA [23]. The peak level of gene expression can be recaptured by periodic plasmid DNA injection [22]. These inherent abilities of the liver provide the solid theoretical basis for our strategy. The liver is the largest internal organ in the body and is responsible for crucial metabolic functions, the kinetic characteristics of siRNA in our study also confirmed that the peak of VEGFR2 siRNA concentration appeared earlier in the liver than in other organs and the concentration peak is highest in the liver. Therefore, the liver was chosen for the study and proven to be modified to secrete siRNA-encapsulated sEVs. The specific mechanism from the hepatocyte uptake and processing of the plasmid DNA to the final secretion of siRNA-encapsulated sEVs is still being explored by our research group.

Our self-assembled VEGFR2 siRNA delivery strategy is highly-efficient compared with both conventional delivery systems and oral TKIs. First, according to the anatomical features of the circulatory system, sEVs secreted by the liver flow into the IVC (the dominating outflow tract of the liver), pass through the heart and ultimately circulate into the lungs. Theoretically, self-assembled VEGFR2 siRNAs can completely aggregate in the lung, thus avoiding decreasing the siRNA concentration caused by systemic blood shunt and hepatic first‐pass clearance. Second, nanoparticles to deliver siRNAs in our strategy were autologously assembled in and secreted by the liver, with suitable diameters matching the vascular fenestrations (approximately 130 nm), which are optimal for avoiding RES clearance and are best suited for intratumoral delivery. As expected, our results proved that the self-assembled VEGFR2 siRNA strategy showed a good therapeutic effect in OS lung metastasis models, and the effect was even better than that of the positive control apatinib. Therefore, reengineering the liver into an intracorporeal arsenal can significantly shorten the siRNA delivery distance to pulmonary lesions as well as increase the biocompatibility of the siRNA delivery vehicle, thus avoiding unnecessary wastage and increasing the therapeutic effects for treating OS metastasis compared with both conventional siRNA delivery strategies and TKI agents.

Drug safety is the top priority in medical treatment. First, in conventional siRNA delivery strategies, synthetic agents, exogenous EVs and viruses are mainly adopted as transfer vehicles in existing siRNA delivery techniques. Knowing that these foreign substances have a great probability to generate immune responses when they are applied in vivo [12, 17, 37], we drove endogenous sEVs to functionally transfer siRNAs in vivo by exploiting their intrinsic features, this natural formulation is entirely not immunogenic and can coexist with the immune system harmoniously. Second, our study observed serious cutaneous reactions and increased serum liver injury markers (ALT, AST, ALP, and γ-GT) over the course of apatinib treatment. Such abnormal serum indicators have often been classified as “drug hepatotoxicity” and may lead to drug discontinuation by clinicians in clinical practice. These inherent tissue damage and toxic adverse effects associated with the mechanism of action seriously hinder the application of TKIs in routine clinical practice. Conversely, anti-VEGFR2 circuit treatment showed nontoxic and safe properties in our research. Therefore, our self-assembled VEGFR2 siRNA strategy may be a safer therapeutic regimen compared with both conventional siRNA delivery strategies and commonly used TKI agents.

Admittedly, the present study still has room for improvement. First, our single-agent/single-target strategy has not yet been assessed for drug resistance. OS is a genetically diverse tumor with profound inter- and intratumor heterogeneity and no specific pattern of tumor genotype [38], thus long-term monotherapy is prone to developing drug resistance in clinical practice. Apart from the VEGF/VEGFR2 pathway, genetic aberrations of the PDGFR, MYC, mTOR, Wnt and PI3K signaling pathways can also serve as therapeutic targets. Multitarget modules can be exploited in genetic circuits to prevent the emergence of therapeutic resistance to VEGFR2 in follow-up research. Second, frequent injection of genetic circuits is temporarily inevitable for long-term treatment, ascribed to the nonpersistent self-assembly and biological effect of siRNAs. Gene editing by CRISPR/Cas9 technology or adeno-associated virus (AAV) can be adopted to solve the problems associated with repeated injections, and corresponding work is underway in our research group. Third, the therapeutic potential and toxic effect have only been validated in the mouse OS lung metastasis model, and more in-depth clinical studies are indispensable for further evaluation of potential clinical utility.

Taken together, this study developed a synthetic biology-based strategy that reprogrammed the host liver to autonomously synthesize VEGFR2 siRNAs to continuously facilitate in vivo siRNA delivery to the lung for gene silencing by secretory sEVs and ultimately achieve antitumor therapeutic effects in a mouse OS pulmonary metastasis model. Our strategy may provide a viable and robust solution for refractory OS lung metastasis and make RNAi therapy feasible for clinical application.

## Materials and methods

### Cell lines

The human umbilical vein endothelial cell line HUVEC (RRID: CVCL_2959, female), mouse endothelial cell line EOMA (RRID: CVCL_3507), and human osteosarcoma cell line 143B (RRID: CVCL_2270, female) were purchased from American Type Culture Collection (ATCC). HUVECs were maintained in endothelial cell medium (ECM, ScienCell, USA) supplemented with 5% fetal bovine serum (FBS, ScienCell, USA) and endothelial cell growth supplement (ECGS, ScienCell, USA). EOMA cells were maintained in Dulbecco’s modified Eagle’s Medium (DMEM) supplemented with 10% FBS (Gibco, Grand Island, NY, USA). 143B cells were maintained in Eagle’s minimum essential medium (EMEM) supplemented with 10% FBS. Primary hepatocytes were isolated from mice as described below and cultured in DMEM supplemented with 10% FBS. All cells were incubated at 5% CO2 at 37 °C. Mycoplasma infection was ruled out in all cell lines using the Venor GeM Mycoplasma Detection Kit (Minerva Biolabs, Berlin, Germany).

### In vivo animal studies

BALB/c nude mice (male, 4 weeks old), NOD/SCID mice (male, 4 weeks old) and C57BL/6 J mice (male, 6 to 8 weeks old) were purchased from the Model Animal Research Centre of Nanjing University (Nanjing, China) from GemPharmatech (Jiangsu, China) and maintained under specific pathogen-free conditions at Nanjing University. The number of mice in each group was chosen according to the calculation formula reported by Charan and Kantharia [39]. In efficacy evaluation in the tail vein metastasis model, 30 NOD/SCID mice for survival assessment were randomly assigned into three group (*n* = 10 in each group) and 18 NOD/SCID mice for tumor evaluation were randomly assigned into three group (*n* = 6 in each group). In efficacy evaluation in the spontaneous metastasis model, 16 NOD/SCID mice for survival assessment were randomly assigned into three group (*n* = 5 in scrRNA and apatinib group, *n* = 6 in anti-VEGFR2 group), and 15 NOD/SCID mice for tumor evaluation were randomly assigned into three group (*n* = 5 in each group). In safety evaluation, 21 C57BL/6 J mice were randomly assigned into three group (*n* = 7 in each group). All mouse studies were conducted in compliance with the Guide for the Care and Use of Laboratory Animals published by the National Institutes of Health and approved by the Animal Ethical and Welfare Committee of Nanjing University (IACUC-2101002-1). All experiments strictly observed the panel’s specific guidelines with respect to the care, treatment and euthanasia of animals used in this study.

### Human studies

A total of 34 surgically resected freshly frozen OS samples (17 primary OS samples and 17 OS lung metastatic samples) were obtained from OS patients at Jinling Hospital, Affiliated Hospital of Medical School, Nanjing University (Nanjing, China). The histopathological diagnosis of the resected samples was confirmed based on the criteria from the World Health Organization (WHO). Informed consent was signed by the participating patients or their legally authorized representatives to utilize their surgical samples, and the study protocol was approved by the Ethics Committee of the said hospital (2021NZKY-040-03). Seventeen paired fresh paired samples (cohorts 1 and 2) were obtained from January 2017 to June 2022. Among these samples, 5 fresh paired samples (cohort 1) were obtained and subjected to IHC assay, and the remaining 12 paired samples (cohort 2) were used for validation of VEGFR2 and CD31 expression. The collection and delivery of clinical samples were performed according to standard processes. After being removed from the patients, the tissue samples were sliced into sections <0.5 cm. The fresh samples were immediately stored in liquid nitrogen and stored frozen until further analysis. Shipments of samples were kept cool with dry ice.

### Design and construction of the genetic circuits targeting VEGFR2

The anti-human VEGFR2 circuit (in the form of naked DNA plasmids) was generated by inserting human VEGFR2 siRNA sequence (5′-CGTTGAGATTTGAAATGGA-3′) into a 171-bp pre-miR-155 backbone with structurally conserved nucleotide substitutions to keep pairing (5′-GGATCCTGGAGGCTTGCTGAAGGCTGTATGCTGAATTCGCGTTGAGATTTGAAATGGAGTTTTGGCCACTGACTGACTCCATTTCAAATCTCAACGCAACACCGGTCAGGACACAAGGCCTGTTACTAGCACTCACATGGAACAAATGGCCCAGATCTGGCCGCACTCGAG-3′) The anti-mouse VEGFR2 circuit was generated by inserting a mouse VEGFR2 siRNA sequence (5′-CATTGAGGTTTGAAATCGACC-3′) using the same approach 5′-GGATCCTGGAGGCTTGCTGAAGGCTGTATGCTGAATTCGCATTGAGGTGAAATCGACCGTTTTGGCCACTGACTGACGGTCGATTTCACCTCAATGCAACACCGGTCAGGACACAAGGCCTGTTACTAGCACTCACATGGAACAAATGGCCCAGATCTGGCCGCACTCGAG-3′. Then the cDNA clone encoding the pre-miR-155 backbone containing either human or mouse VEGFR2 siRNA was inserted downstream of the CMV promoter. A circuit constructed to express a scrambled RNA (scrRNA) using the same approach was used as the negative control. The siRNAs were synthesized by RiboBio (Guangzhou, China). The genetic circuits were synthesized by GenScript (Piscataway, NJ, USA). The scaffold and map of the genetic circuit are shown in Fig. S9.

E. coli DH5α competent cells (Tsingke, TSC01, Beijing, China) were applied for plasmid transformation, and then the cultures were inoculated in LB medium supplemented with 50 μg/mL spectinomycin (Solarbio, China) and inoculated overnight on a shaker table at 220 rpm and 37 °C. Fourteen hours later, plasmids were extracted using an EndoFree Maxi Plasmid Kit V2 (Tiangen, DP120, Beijing, China) according to the manufacturer’s instructions. The correctness of the inserted sequences in the purified plasmids was eventually verified by Sanger sequencing.

### Extraction and identification of primary hepatocytes

Primary hepatocytes were collected from male C57BL/6 J mice aged 6-8 weeks according to a shared protocol online (http://mouselivercells.com/). Briefly, midline laparotomy was performed under anesthesia and the hepatic portal vein was exposed. A 24-G cannula was introduced into the portal vein and secured using a hemostatic clip. The IVC was transected to allow outflow of perfusate. The liver was sequentially perfused with the following solutions at a flow rate of 5 ml/min, first with 50 ml of calcium- and magnesium-free HBSS (CMF-HBSS, Solarbio, China), followed by 50 ml of HBSS (Solarbio, China) plus 0.05% collagenase Type IV (Yeasen, Shanghai, China). All solutions were warmed to 37 °C. After perfusion, the lobes of the liver were transferred into a Petri dish containing DMEM with 10% FBS to disperse the hepatocytes through a 70-μm nylon mesh. The hepatocyte slurry was transferred to a 50 ml centrifuge tube following centrifugation at 50 g for 3 min. The hepatocyte pellet was gently resuspended in 5 ml complete DMEM medium and 5 ml 100% percoll (nine-tenth Percoll plus one-tenth 9% NaCl). The mixture was centrifuged at 200 g for 5 min with no brake. The hepatocyte pellet was washed with PBS as above and then seeded with complete DMEM medium at 5% CO2 at 37 °C. The primary hepatocytes were characterized by the specific marker ALB (RRID: AB_11042320), and the hepatocyte-specific morphology and ALB expression level were verified by immunofluorescence using a Zeiss LSM 880 confocal microscope (Carl Zeiss, Oberkochen, Germany).

### In vivo injection of the genetic circuits

Male C57BL/6 J mice aged 6-8 weeks (Model Animal Research Centre of Nanjing University, Nanjing, China) were maintained under specific pathogen-free conditions. The genetic circuits (in the form of plasmids) were administered to mice at a concentration of 10 mg/kg via regular tail vein IV injection at approximately 200 μl per mouse every two days. The number of injections was dependent on the duration of different experiments. Our injection strategy is three times in in vivo fluorescence tracing of sEVs, seven times in all the other experiments. After injection, the mice were sacrificed, blood samples were collected through cardiac puncture, and various organs were harvested.

### Quantitative reverse transcription PCR (qRT-PCR)

Total RNA was isolated from mouse tissues or cultured cells using TRIzol (Invitrogen, Carlsbad, CA, USA) according to the manufacturer’s instructions.

#### siRNA detection

Mature VEGFR2 siRNA was detected using a miRNA 1st Strand cDNA Synthesis Kit (by stem-loop) and miRNA Universal SYBR qPCR Master Mix (Vazyme, Nanjing, China). The reverse transcription parameters were as follows: 25 °C for 5 min, 50 °C for 15 min and 85 °C for 5 s. The amplifications were incubated at 95 °C for 5 min and then subjected to 45 cycles of 95 °C for 10 s and 60 °C for 30 s. For quantification of the relative amounts of VEGFR2 siRNAs in sEVs, endogenous miR-16 was used for normalization. For quantification of the absolute levels of VEGFR2 siRNAs, single-stranded VEGFR2 siRNA synthesized by GenScript (Piscataway, NJ, USA) was serially diluted to construct a standard curve, and a no-template control was calculated simultaneously to verify primer specificity. By reference to the qRT-PCR standard curve, the concentrations of VEGFR2 siRNA in sEVs, serum components and different tissues were assessed and presented as the absolute amounts of VEGFR2 siRNA in 1 L of serum (fmol/L) or 1 g of total RNA (pmol/g total RNA). In miRNA-cDNA synthesis, specific stem-loop primers for human VEGFR2 siRNA (5′-GTCGTATCCAGTGCAGGGTCCGAGGTATTCGCACTGGATACGACTCCATT-3′), mouse VEGFR2 siRNA (5′-GTCGTATCCAGTGCAGGGTCCGAGGTATTCGCACTGGATACGACGGTCGA-3′), miR-16 (5′-GTCGTATCCAGTGCAGGGTCCGAGGTATTCGCACTGGATACGACCGCCAA-3′) were used. In the miRNA qPCR assay, specific forward primers for human VEGFR2 siRNA (5′-GCGCGCGTTGAGATTTGA-3′), mouse VEGFR2 siRNA (5′-CGCGCATTGAGGTTTGAAA-3′), miR-16 (5′-CGCGTAGCAGCACGTAAATA-3′), and universal reverse primer (5′-AGTGCAGGGTCCGAGGTATT-3′) were used.

#### mRNA detection

For VEGFR2 mRNA analysis, HiScript II Q Select RT SuperMix and ChamQ Universal SYBR qPCR Master Mix (Vazyme, Nanjing, China) were applied. The RT-PCR was performed as follows: 50 °C for 15 min and 85 °C for 5 s. The amplification was performed by predenaturation at 95 °C for 30 s, followed by 40 cycles of 95 °C for 10 s and 60 °C for 30 s. All reactions were run in triplicate using a LightCycler96 System (Roche, IN, USA). After completion of the reactions, the relative fold-change of mRNA was normalized to human or mouse GAPDH and analyzed by the 2^−△△CT^ method. Specific primers were used for human VEGFR2 (forward: 5′-GTGACCAACATGGAGTCGTG-3′; reverse: 5′-TGCTTCACAGAAGACCATGC-3′), mouse VEGFR2 (forward: 5′-ACCAGAAGTAAAAGTGATCCCAGA-3′; reverse: 5′-TCCACCAAAAGATGGAGATAATTT-3′), human GAPDH (forward: 5′-GCTCTCTGCTCCTCCTGTTCG-3′; reverse: 5′-GCGAACACATCCGGCCTGC-3′), and mouse GAPDH (forward: 5′-ACGGCAAATTCAACGGCAC-3′; reverse: 5′-TAGTGGGGTCTCGCTCCTGG-3′). All primers were synthesized by GenScript (Piscataway, NJ, USA).

### Western blotting

Cells and tissue samples were lysed in RIPA Lysis Buffer (Beyotime, Shanghai, China) supplemented with Protease inhibitor cocktail for general use (Beyotime, Shanghai, China) on ice for 30 min. Cell lysates and tissue homogenates were centrifuged at 4 °C (12,000 × g for 10 min), and the supernatant was collected. The protein concentration was determined using a Pierce BCA Protein Assay kit (Thermo Scientific, CA, USA). Protein expression levels were verified using western blotting with corresponding antibodies. Briefly, protein was electrophoretically separated and transferred onto polyvinylidene fluoride (PVDF) membranes (Millipore, Billerica, USA). The membranes were blocked for 15 min with Blocking Buffer for Western Blot (Beyotime, Shanghai, China) and incubated overnight at 4 °C with primary antibodies. After 3 washes with 1 × Tris-buffered saline with 0.1% Tween-20 (TBST) for 10 min per wash, the membranes were incubated with secondary antibodies for 1 h at room temperature and then subjected to three additional 15-min washes with 1 × TBST. The membranes were incubated in a Dura ECL kit (Fudebio, Hangzhou, China) and visualized by a chemiluminescence system (Tanon, Shanghai, China). The primary antibodies were as follows: anti-Alix (RRID: AB_673819), anti-TSG101 (RRID: AB_2208090), anti-CD9 (RRID: AB_627213), anti-AGO2 (RRID: AB_2096299), anti-VEGFR2 (RRID: AB_2212507), anti-CD31 (RRID: AB_2160882), and anti-β-Actin (RRID: AB_10950489). The secondary antibodies were HRP-conjugated AffiniPure goat anti-rabbit IgG (H + L) (RRID: AB_2722564) and HRP-conjugated AffiniPure goat anti-mouse IgG (H + L) (RRID: AB_2722565).

### Immunohistochemistry (IHC)

The paraformaldehyde-fixed and paraffin-embedded tissues were immunostained with anti-Ki-67 (RRID: AB_393778), anti-E-cadherin (RRID: AB_2291471), anti-N-cadherin (RRID: AB_2813891), anti-vimentin (RRID: AB_2273020), anti-CD31 (RRID: AB_2923131), and anti-VEGFR2 (RRID: AB_2212507) antibodies at 4 °C overnight, and a two-step immunohistochemistry kit (Zsgb-bio, Beijing, China) was used as appropriate. After incubation using a DAB Substrate Kit (Abcam, USA), the staining was visualized. Finally, the relative expression level was assessed via the percentage of positive areas.

### Small extracellular vesicle (sEV) isolation

Isolation and purification of sEVs from both cell culture supernatants and serum were performed using ultracentrifugation according to the previous literature [40].

#### sEV isolation from cell culture supernatants

Before isolating the sEVs from cell culture supernatants, FBS-derived sEVs from FBS-containing medium were pre-depleted to obtain FBS-sEV-free medium. Briefly, medium supplemented with 20% FBS was prepared and centrifuged using a Beckman Optima L-100 XP ultracentrifuge (100,000 g overnight, 4 °C). Then the supernatant was filter sterilized through a 0.22 μm filter and diluted to a final FBS concentration (10%) to make the sEV production medium. When the primary hepatocytes reached 70–80% confluency, cell culture supernatants were collected. Then, the conditioned medium was subjected to successive centrifugation at increasing speeds to eliminate large dead cells and large cell debris (300 g, 10 min, 4 °C, followed by 2000 g, 10 min, 4 °C, eventually 10,000 g, 30 min, 4 °C). The supernatant was transferred to a fresh ultracentrifuge tube and subjected to ultracentrifugation (100,000 g, 90 min, 4 °C) to collect the sEV fraction. The sEV pellet was resuspended in PBS and ultracentrifuged again (100,000 g, 70 min, 4 °C) to wash the sEVs. The resulting sEVs were responded to in sterile PBS for subsequent experiments.

#### sEV isolation from serum

Blood samples harvested from mice injected with genetic circuits (plasmids) were tranquillized for 30 min and then centrifuged at 3000 g for 15 min to remove cells and cell debris. After centrifugal stratification, the obtained serum was diluted with an equal volume of PBS, and the diluted serum was centrifuged at 2000 g for 30 min at 4 °C, followed by 12,000 g for 45 min at 4 °C to eliminate large dead cells and cell debris. Then, the supernatant was ultracentrifuged at 110,000 g for 2 h at 4 °C, and the pellets were resuspended in PBS and filter sterilized through a 0.22 μm filter. The resuspension was subjected to ultracentrifugation twice at 110,000 g for 70 min at 4 °C to obtain the resulting serum-derived sEVs.

### sEV analysis

#### Transmission electron microscopy (TEM)

The purified sEVs were visualized by an H-7650 Hitachi TEM (Zhongjingkeyi Technology Co., Ltd., Beijing, China). Briefly, sEVs resuspended in PBS were fixed in 2% paraformaldehyde and then adsorbed onto a formaldehyde-coated copper mesh in a dry environment for 20 min. The sample was fixed in 1% glutaraldehyde for 5 min. After rinsing in distilled water, the sample was dyed with uranyl oxalate for 5 min and then dyed with uranyl acetate for 10 min on ice. Excess liquid was removed from the mesh with filter paper, and the mesh was stored at room temperature until imaging.

#### Nanoparticle tracking analysis (NTA)

The particle size distribution and quantity of sEVs were analyzed using a NanoSight NS 300 system and NTA 3.2 Software (NanoSight, Wiltshire, UK). The nanoparticles were illuminated with the blue laser, and their Brownian motion was captured for 60 s. At least 5 videos were collected from each individual sample to provide representative concentration measurements. The size distribution curves were evaluated with NTA software and averaged within each sample from the video repetitions and then averaged between repetitions to provide a representative size distribution.

### Magnetic endothelial cells separation

Dynabeads conjugated with the anti-CD31 antibody (RRID: AB_2722705) were prepared using the Dynabeads Antibody Coupling Kit (Life Technologies, 14311D) following the manufacturer’s instructions. Mouse lung tissues were obtained immediately after sacrifice, and were washed multiple times with 1X PBS buffer (approximately 3 to 6 times). The lung tissues were then minced into 1–2 mm pieces using a scalpel in the presence of 0.25% collagenase I (Yeasen, Shanghai, China) solution (in PBS + 20% FBS) and were digested in the same solution for 45 min in a 37 °C shaking incubator. After digestion, the cells were filtered through a sterile 70 μm nylon mesh and were washed with cold 1X PBS buffer. The cells were then incubated with the conjugated dynabeads for 30 min at 4 °C to allow binding. The bead-bound cells were washed four times with 1X PBS buffer and collected using a magnet. Finally, all the bead-bound cells were resuspended in DMEM with 20% FBS before being plated.

### *sEV* incubation assay

C57BL/6 J mice were injected IV with a genetic circuit (10 mg/kg) every 2 days for a total of 7 times, and then the sEVs from serum or primary hepatocytes were purified as described above. A fluorescence colocalization experiment was performed according to the manufacturer’s manual. Briefly, 100 μg total protein content of sEVs (approximately 2.85 × 10^10^ sEVs) was monitored using the membrane cell marker PKH26 (Sigma-Aldrich) and incubated with 1 × 10^6^ HUVECs for 6 h. Then, the colocalization of the sEVs with HUVECs was verified by immunofluorescence using a Zeiss LSM 880 confocal microscope (Carl Zeiss, Oberkochen, Germany).

For primary hepatocyte and serum-derived sEV incubation, different doses of sEVs (total protein contents were 4, 20 and 100 μg) were incubated with 1 × 10^6^ HUVECs for 36 h, and then total RNA or protein was isolated for knockdown validation. According to the results of western blotting and qRT-PCR, 100 μg total protein content of sEVs (approximately 2.85 × 10^10^ sEVs) was selected as the appropriate dose and then incubated with 1 × 10^6^ HUVECs for functional verification.

### Cell Counting Kit (CCK)-8 assay

For the CCK-8 assay, HUVECs were equally seeded in 96-well cell culture plates (3 × 10^3^ cells per well) and incubated with serum sEVs purified from mice injected with genetic circuits (plasmids) at the appropriate concentrations as described above. At different time points (0, 24, 48, 72 h and 96 h), another 100 μL of complete medium containing 10 μL of CCK-8 (APExBio, USA) and serum sEVs was added to the culture plates and incubated for 1 h. The absorbance at 450 nm was detected using a microplate reader (Thermo Scientific, CA, USA).

### 5-Ethynyl-2′-deoxyuridine (EdU) assay

An EdU assay kit was purchased from RiboBio (RiboBio Inc., Guangzhou, China) for EdU assays. HUVECs were seeded into 96-well cell culture plates and incubated with serum sEVs overnight. On the following day, EdU solution (25 μM) was added to the complete medium and incubated for the indicated time periods. Cells in the culture plates were fixed with paraformaldehyde for 2 h and permeabilized with 0.5% Triton X-100 for 10 min. Apollo reaction solution (200 μL) and DAPI (200 μL) were added to stain EdU and nuclei, respectively, for 30 min. Luminescence was photographed using an Olympus IX 71 inverted fluorescence microscope (Olympus, Tokyo, Japan) to analyze cell proliferation and DNA synthesis. For image quantification, at least 5 randomly selected images were chosen from each independent experiment, and the mean level of 3 independent experiments was quantified.

### Wound healing assay

HUVECs were seeded into 6-well cell culture plates and incubated with serum sEVs until 100% confluence. Then, the cells were scraped off with a 200 μl pipette tip and seeded in serum-free medium containing serum sEVs. Wound closure was photographed with an Olympus IX 71 inverted fluorescence microscope (Olympus, Tokyo, Japan) at 0 and 24 h after scratching.

### Transwell migration assay

The Transwell chamber was purchased from Corning Costar (NY, USA) for migration verification. Briefly, medium supplemented with 20% FBS and serum sEVs was added to the lower chambers as a chemoattractant, and 5 × 10^4^ HUVECs were suspended in serum-free medium and added to the upper chambers. After 24- h of coculture, the cells in the filter were fixed and then stained with 0.1% crystal violet; migrated cells were photographed and counted using an Olympus IX 71 inverted fluorescence microscope (Olympus, Tokyo, Japan). For image quantification, at least 5 randomly selected images were chosen from each independent experiment, and the mean level of 3 independent experiments was quantified.

### Tube formation assay

An in vitro tube formation assay was performed as previously described [41]. Briefly, Matrigel (BD Sciences, MA, USA) was coated on 96-well culture plates and homogenized at 37 °C for 30 min until gelation. Then, HUVECs (15 × 10^3^ cells per well) were seeded into culture plates, incubated with serum sEVs for 6 h, and stained with calcein-AM (Yeasen, Shanghai, China). Green fluorescence was photographed with an Olympus IX 71 inverted fluorescence microscope (Olympus, Tokyo, Japan), and total tube formation was analyzed by the ImageJ plug-in for angiogenesis (NIH, http://rsb.info.nih.gov/ij/). For image quantification, at least 3 randomly selected images were chosen from each independent experiment, and the mean level of 3 independent experiments was quantified.

### Aortic ring assay

The aortic ring assay was performed according to a previous report [42]. In brief, thoracic aortas were excised from male C57BL/6 J mice aged 6–8 weeks and sliced into 1 mm sections. The rings were embedded per well of 96-well culture plates containing 50 μL of collagen type I (Millipore, Billerica, MA, USA), and sEVs were incubated under sterile conditions. After 7 days of incubation, the aortic ring was fixed with paraformaldehyde, specifically stained with fluorescent antibody, and finally photographed using a Zeiss LSM 880 confocal microscope (Carl Zeiss, Oberkochen, Germany). The microvessel area was quantified by TRI2 (http://www.assembla.com/spaces/ATD_TRI/wiki). The reagents and antibodies used were as follows: BS1 lectin-FITC (Sigma-Aldrich, L9381), anti-α-SMA (RRID: AB_2811044), and Donkey anti-Rabbit IgG (H + L) Highly Cross-Adsorbed Secondary Antibody, Alexa Fluor™ 594 (RRID: AB_141637).

### Chick embryo chorioallantoic membrane (CAM) assay

We performed a CAM assay based on published literature [43]. After 48 h of incubation, 2–3 ml albumen was aspirated, and then a square window was cut on the shell of the fertilized sterile egg (Boehringer Ingelheim, Beijing, China). After sealing the window with transport tape, the egg was incubated at 37 °C for another 6 days. At day 8 of incubation, the window was opened, and a sterilized gelatin sponge containing serum sEVs was placed onto the CAM for the CAM assay by using at least 6 eggs for each group. The egg was incubated until day 12, and the blood vessel areas on the CAM were quantitatively analyzed according to the published literature [44] using ImageJ software. The branching points were analyzed by the ImageJ plug-in for angiogenesis.

### In vivo therapeutic efficacy evaluation in the OS metastasis model

To generate the tail vein OS lung metastasis model, a total of 5 × 10^6^ luminescence-labeled 143B cells were suspended in PBS and injected IV into BALB/c nude male mice aged 4 weeks via the tail vein. Four weeks after injection, lung metastasis was assessed by an IVIS Spectrum In Vivo Imaging System (PerkinElmer, USA), and the specific location and size of the metastatic tumors were analyzed by micro-CT (Hiscan, Suzhou, China). To generate the spontaneous OS metastasis model, a total of 5 × 10^6^ luminescence-labeled 143B cells were suspended in PBS followed by tibial bone marrow cavity injection into NOD/SCID male mice aged 4 weeks using a microliter syringe (Hamilton, Bonaduz, Switzerland). Thirty days after injection, the tumor-bearing right legs of all mice were amputated aseptically. The nude mice and NOD/SCID mice (Model Animal Research Centre of Nanjing University, Nanjing, China) were maintained under specific pathogen-free conditions. Then, the tumor-bearing BALB/c nude mice or amputated NOD/SCID mice were randomly divided into 3 groups: two were injected IV with 10 mg/kg scrRNA circuit or anti-VEGFR2 circuit, and one was intragastrically administered 200 mg/kg apatinib (Abcam Inc., Cambridge, MA, USA) every two days over the course of 2 weeks. Apatinib was selected as a positive control because it has been conclusively proven efficient in anti-angiogenesis and osteosarcoma lung metastasis treatment [7].

After genetic circuits or apatinib treatment, the mice were divided into two groups for survival time monitoring and tumor growth evaluation. For survival analysis, the mice were monitored for survival post circuit injection or apatinib administration without any further treatment. For tumor growth analysis, lung metastasis was assessed by IVIS (PerkinElmer, USA) pre- and posttreatment with the genetic circuits or apatinib. Mice that survived the 2-week treatment were simultaneously analyzed using micro-CT (Hiscan, Suzhou, China). After IVIS detection and micro-CT scanning, the mice were euthanized, and the tumor-bearing lungs were harvested and analyzed using histological analysis (H&E staining). OS lung lesions were also excised and sent for further western blotting and immunohistochemical analyses.

### In vivo biochemical indexes and toxicity evaluation in the OS metastasis model

C57BL/6 J mice were injected IV with 10 mg/kg scrRNA circuit or anti-VEGFR2 circuit or intragastrically administered 200 mg/kg apatinib every 2 days for a total of 7 times. Twelve hours after the last administration, the mice were sacrificed to collect peripheral blood and various tissues. Fresh blood samples were divided into two parts for blood routine indexe and blood biochemical indexe analyses. For routine blood index analysis, peripheral blood was collected in an EDTA anticoagulant vacuum tube. Then, the total number of white blood cells (WBCs), red blood cells (RBCs) and platelets (PLTs) was measured with a hematology analyzer (BC-2800Vet, Mindray, China). For biochemical index analysis, peripheral blood was subjected to serum extraction, and representative serum biochemical parameters were tested, including liver injury indicators including alanine transferase (ALT), aspartate aminotransferase (AST), alkaline phosphatase (ALP), gamma-glutamyltransferase (γ-GT), total bilirubin (TBIL) and direct bilirubin (DBIL); kidney injury indicators including serum creatinine (CREA) and blood urea nitrogen (BUN); and cardiac injury indicators including creatine kinase (CK) and lactate dehydrogenase (LDH). These biochemical indexes were tested with a biochemistry analyzer (Chemray-800, Rayto, China) according to the manufacturer’s instructions. Skin, heart, liver, lung and kidney tissues were extracted for H&E staining and TUNEL staining to assess tissue damage. TUNEL apoptosis detection kit (Alexa Fluor 488) was purchased from Beijing Solarbio Science & Technology Co., Ltd. (Beijing, China) and the experiment was carried out according to the manufacturer’s instructions. Representative sections were photographed using an Olympus IX 71 inverted fluorescence microscope (Olympus, Tokyo, Japan).

### Qualification and statistical analysis

All statistical analyses were conducted using GraphPad Prism 9.20 (GraphPad, Inc., La Jolla, CA). All data are presented as the mean value ± standard deviation (SD) from at least three independent experiments. All the data were assessed by two individuals who were ignorant of the clinical sample information, experimental treatment and animals’ grouping. No samples or animals were excluded from the analysis. Shapiro-Wilk method was used to estimate the normal distribution of data and Levene method was used to test the homogeneity of variance. Two-group comparisons were calculated by Student’s *t*-test; multiple group comparisons were analyzed by one-way ANOVA followed by Bonferroni’s multiple comparisons test. Significance was assumed with *P < 0.05; **P < 0.01; ***P < 0.001; ****P < 0.0001; ns = not significant.

### Supplementary information

Supplementary Information

Raw data of WB

Reproducibility checklist

## Data Availability

The authors declare that all data supporting the findings of this study are available within the paper. Any additional information required to reanalyze the data in this paper is available from corresponding authors upon request.
