# Supplementary material for: In vivo self-assembly and delivery of VEGFR2 siRNA-encapsulated small extracellular vesicles for lung metastatic osteosarcoma therapy
Source: Cell Death Dis. 2023 Sep 22;14(9):626. doi: 10.1038/s41419-023-06159-3 (PMC10516902; doi:10.1038/s41419-023-06159-3)
Supplement: Supplementary file 1 — Supplementary Information [file 41419_2023_6159_MOESM1_ESM.pdf]

In vivo self-assembly and delivery of VEGFR2 siRNA-encapsulated small extracellular vesicles for lung metastatic osteosarcoma therapy

Supplementary Information

Lingfeng Yu†, Gentao Fan†, Qingyan Wang, Yan Zhu, Hao Zhu, Jiang Chang, Zhen Wang, Shoubin Zhan, Xianming Hua, Diankun She, Jianhao Huang, Yicun Wang, Jianning Zhao, Chen-Yu Zhang*, Xi Chen*, Guangxin Zhou*

This PDF file includes:

Figures S1 to S9

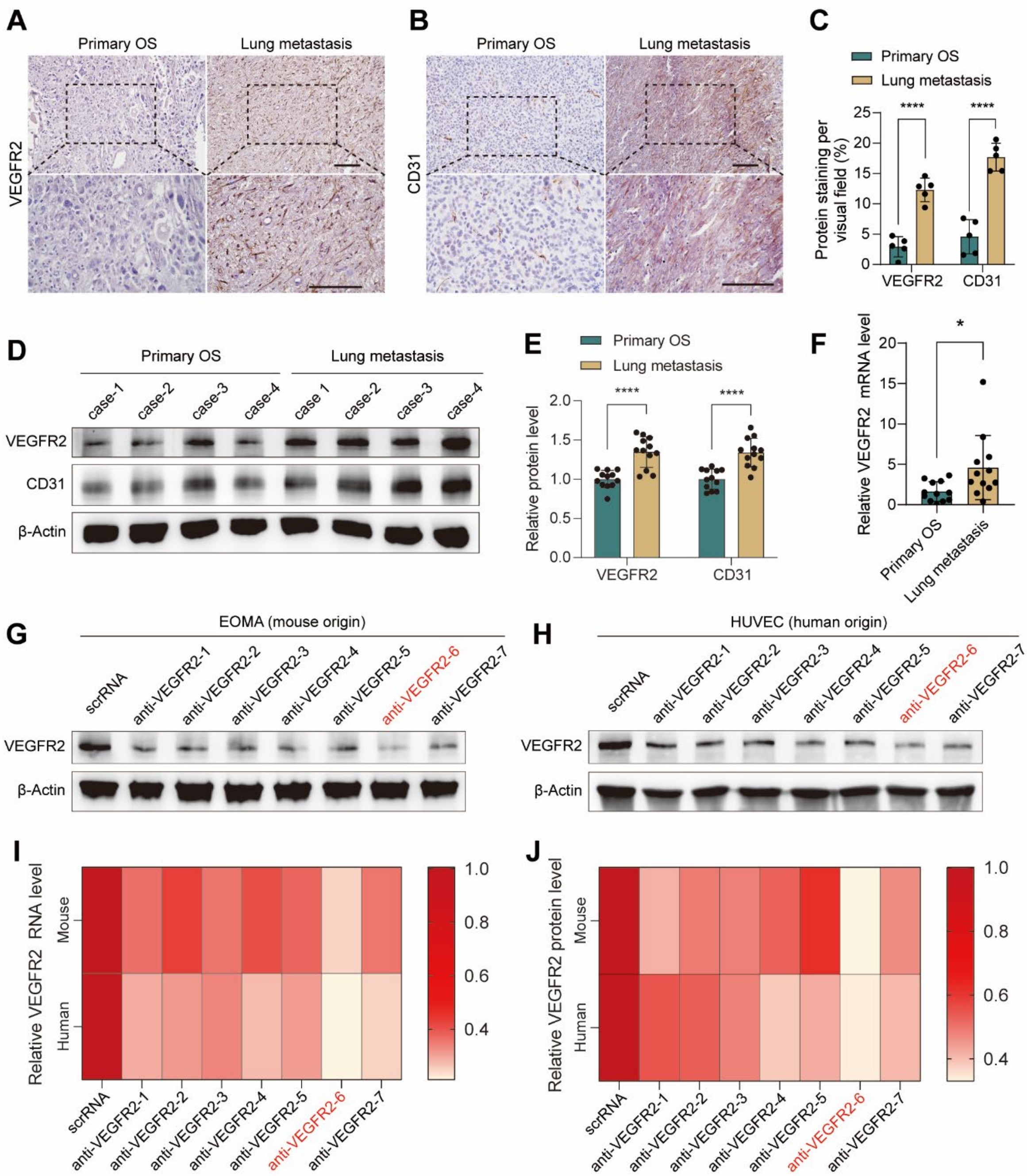

Figure S1. VEGFR2 expression is elevated in clinical osteosarcoma lung metastases.

(A-B) Representative images of IHC staining for VEGFR2 and CD31 proteins in clinical primary OS and lung metastasis sections. Scale bar: 100 µm. **(C)** Quantitative analysis of IHC staining for VEGFR2 and CD31 proteins in clinical primary OS and lung metastatic sections (n = 5 per group). **(D)** Western blot analysis of VEGFR2 and CD31 protein levels in clinical primary OS and lung metastatic sections. Representative western blots from 4 independent clinical samples are shown. **(E)** Quantitation of VEGFR2 and CD31 protein levels in clinical primary OS and lung metastatic sections (n = 12 per group). **(F)** qRT-PCR analysis of VEGFR2 mRNA levels in clinical primary OS and lung metastatic sections (n = 12 per group). **(G-H)** Western blot analysis of the knockdown effect of candidate VEGFR2 siRNAs of human origin **(G)** and mouse origin **(H)** in anti-VEGFR2 circuits. Representative western blots are shown.

(I-J) Heat-maps showing the quantitation of VEGFR2 protein levels **(I)** and mRNA levels **(J)** under the knockdown of candidate anti-VEGFR2 circuits of human origin and mouse origin (n = 3 per group). Values are presented as the mean ± SD. Significance was determined using a two-sided t-test in **F** or using one-way ANOVA in **C** and **E**. *P < 0.05; ****P < 0.0001.

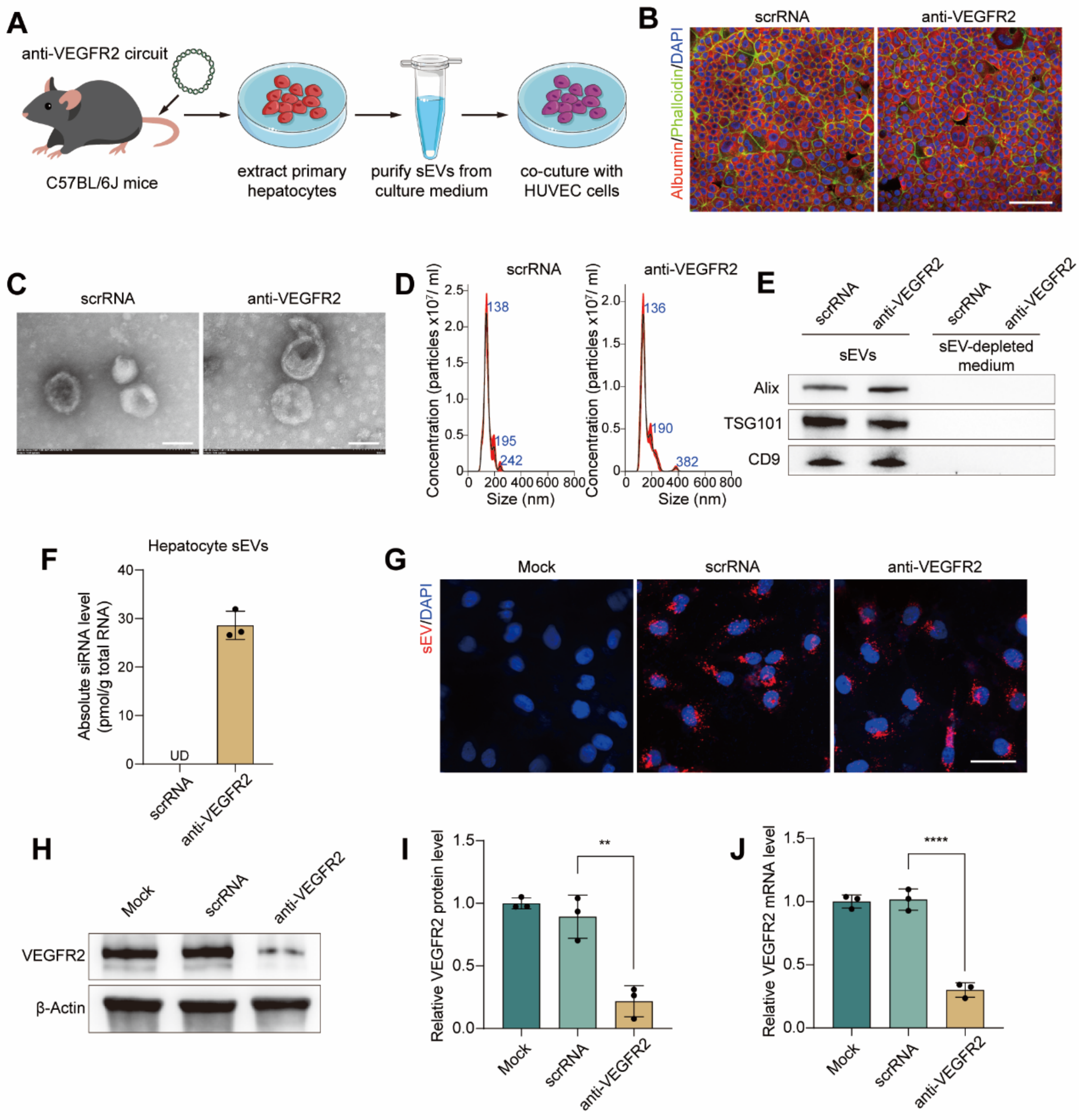

Figure S2. Characterization of self-assembled VEGFR2 siRNA secreted by primary hepatocytes.

(A) Schematic of the experimental design. C57BL/6J mice were intravenously injected with scrRNA circuit or anti-VEGFR2 circuit (10 mg/kg) every 2 days for a total of seven times, and then the primary hepatocytes were extracted from the mouse livers. sEVs purified from the primary hepatocyte culture supernatant were incubated with HUVECs. Next, the uptake of self-assembled VEGFR2 siRNA by HUVECs and the subsequent suppression of VEGFR2 expression by self-assembled VEGFR2 siRNA were examined in this *ex vivo* model. **(B)** Representative images of immunofluorescence staining for primary hepatocytes. Hepatocytes showed strong staining for albumin (red); phalloidin (green)-stained cellular cytoskeleton; DAPI-stained nuclei (blue). Scale bar: 100 μm. **(C)** Representative TEM images of hepatocyte-secreted sEVs. Scale bar: 100 nm. **(D)** The size distribution and concentration of hepatocyte-secreted sEVs were determined by NTA. **(E)** Western blot analysis of specific markers (Alix, TSG101 and CD9) in hepatocyte-secreted sEVs and sEV-depleted culture supernatant. **(F)** qRT-PCR analysis of VEGFR2 siRNA levels in hepatocyte-secreted sEVs (n = 3 per group). UD, undetected. **(G)** Hepatocyte-secreted sEVs were fluorescently labeled with PKH26, and PKH26-labeled sEVs were incubated with HUVECs for 6 h. The levels of intracellular fluorescence intensity were monitored by confocal microscopy. Scale bar: 50 μm. **(H-I)** Western blot analysis of VEGFR2 protein levels in HUVECs after 36 h of incubation with hepatocyte-secreted sEVs. Representative western blots **(H)** and densitometric analysis data **(I)** are shown (n = 3 per group). **(J)** qRT–PCR analysis of VEGFR2 mRNA levels in HUVECs after 36 h of incubation with hepatocyte-secreted sEVs (n = 3 per group). Values are presented as the mean ± SD. Significance was determined using a two-sided t-test in **I** and **J**. $^{**}P < 0.01$; $^{****}P < 0.0001$.

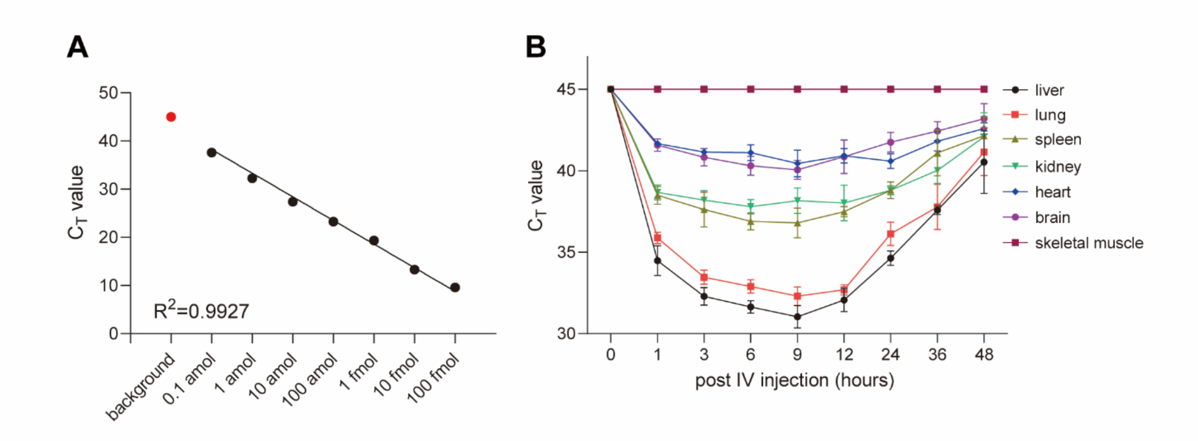

Figure S3. Tissue distribution kinetics of VEGFR2 siRNA in multiple mouse tissues followed by tail vein injection of the genetic circuit.

(A) The sensitivity and dynamic range of the qRT-PCR assay for measuring VEGFR2 siRNA. Synthetic single-stranded VEGFR2 siRNA was serially diluted over several orders of magnitude (ranging from 0.1 attomole to 1 femtomole). The resulting C_T values assessed via qRT-PCR were plotted against the corresponding amount of input VEGFR2 siRNA to generate a standard curve. A "no template background" (water) was used in place of siRNA to determine the specificity of the primer set. Synthetic VEGFR2 siRNAs were efficiently amplified at C_T values ranging from 9.59 to 37.59 in a linear fashion, while the water control was not adequately amplified (C_T value = 45). **(B)** The individual C_T values for VEGFR2 siRNA in various mouse tissues followed by tail vein injection of 10 mg/kg anti-VEGFR2 circuit (n = 3 per group). The C_T values of VEGFR2 siRNA in the liver, lung, spleen, kidney, heart and brain were consistently within the linear range at all time points but were outside the linear range for skeletal muscle, as illustrated in the individual C_T- vs.- time curve. By referring to the standard curve, the concentration of VEGFR2 siRNA in various tissues was calculated.

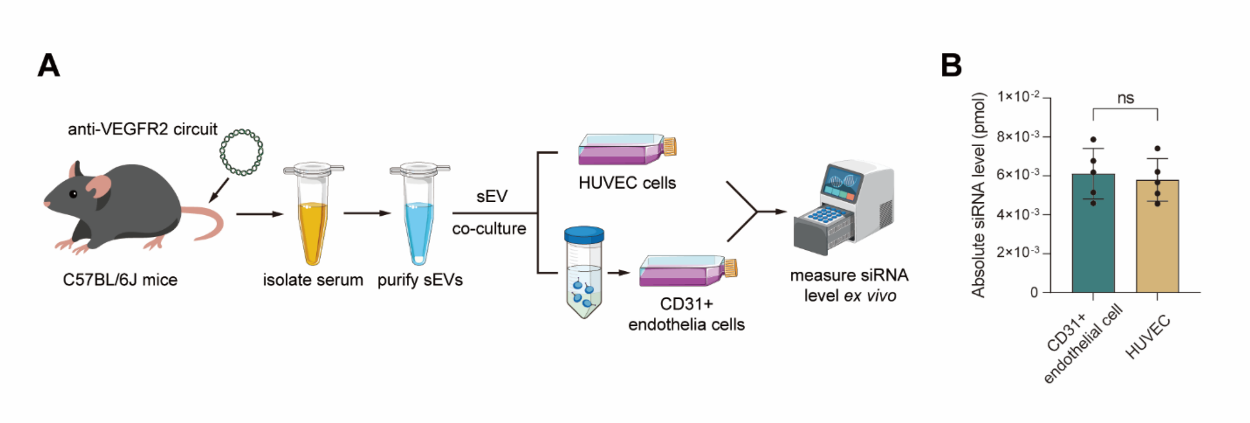

Figure S4. Evaluation of VEGFR2 siRNA enrichment in HUVECs and CD31+ pulmonary microvascular endothelial cells.

(A) Schematic of the experimental design. Serum sEVs (100 µg total protein) were harvested from C57BL/6J mice injected with anti-VEGFR2 circuit (10 mg/kg) for a total of seven times, and were subsequently subjected to evaluation of the VEGFR2 siRNA enrichment level by incubating with HUVECs (100,000 cells) and CD31+ pulmonary microvascular endothelial cells (100,000 cells).

(B) qRT–PCR analysis of VEGFR2 siRNA levels in HUVECs and CD31+ endothelial cells after 36 h of incubation with sEVs (n = 5 per group). Values are presented as the mean ± SD. Significance was determined using a two-sided t-test in **B**. ns = not significant.

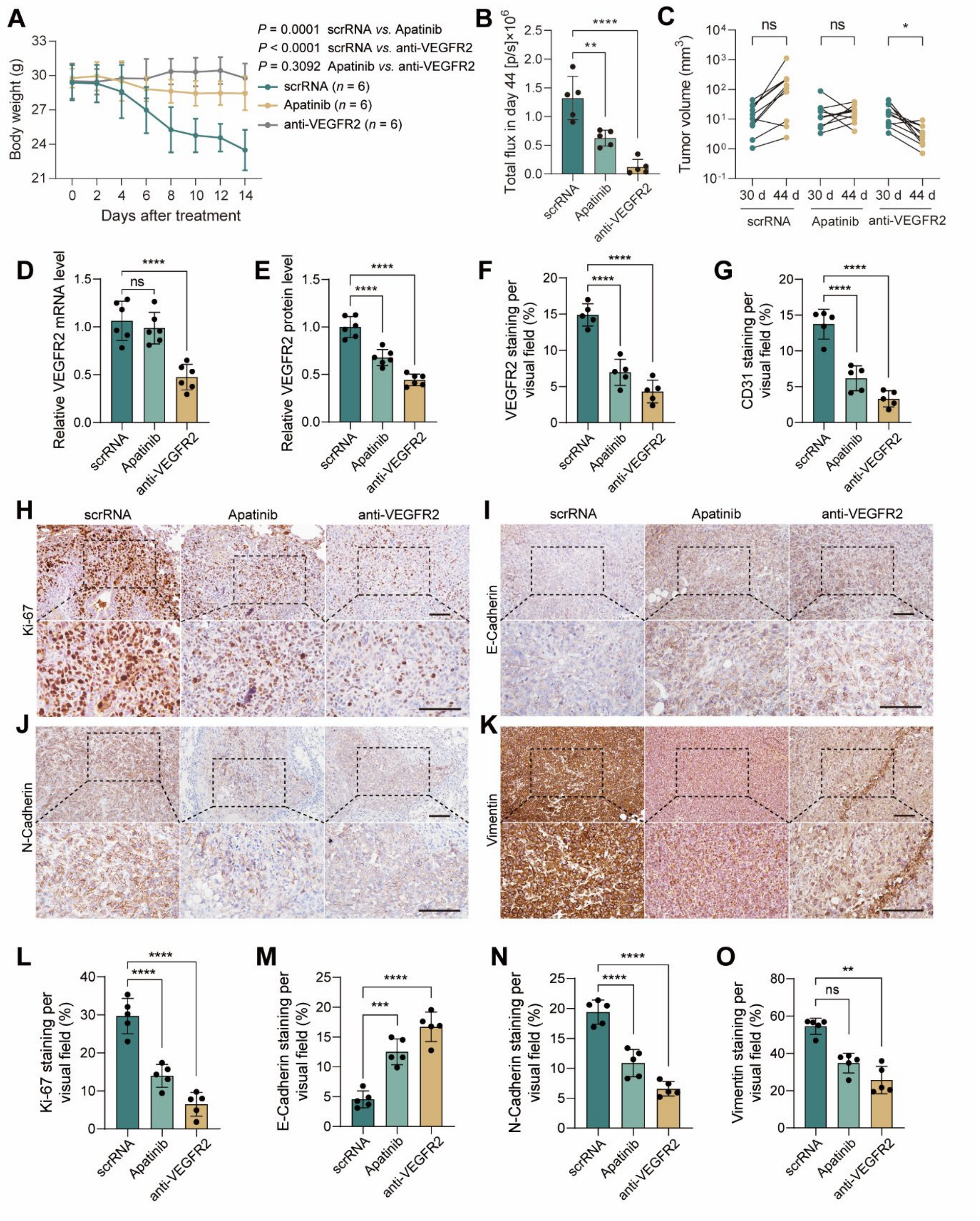

Figure S5. Evaluation of the therapeutic efficacy of self-assembled VEGFR2 siRNA in the tail vein OS metastasis model.

Nude mice were intravenously injected with stable 143B cells labeled with firefly luciferase to construct OS lung metastatic model. Thirty days after injection, the tumor-burdened mice were divided into two groups for either survival analysis or tumor evaluation and then were intravenously injected with the scrRNA circuit or anti-VEGFR2 circuit (10 mg/kg) or intragastrically administered 200 mg/kg apatinib every 2 days for a total of seven treatments. After treatment, body weight, tumor growth and VEGFR2 expression levels were evaluated. **(A)** Body weight curves (n = 6 per group). **(B)** Luminescence intensity quantification of 143B cells in the lung from mice posttreatment (day 44) with the genetic circuits or apatinib (n = 5 per group). **(C)** Semiautomated quantitative image analysis of tumor volumes pre- and posttreatment with genetic circuits or apatinib using 3-D reconstructions (n = 10 per group). **(D)** Quantitation of VEGFR2 mRNA levels in mouse lung metastatic

samples (n = 6 per group). **(E)** Quantitation of VEGFR2 protein levels in mouse lung metastatic samples (n = 6 per group). **(F-G)** Quantitative analysis of IHC staining for VEGFR2 and CD31 proteins in mouse lung metastatic samples (n = 5 per group). **(H-K)** Representative images of IHC staining for Ki-67 **(H)**, E-cadherin **(I)**, N-cadherin **(J)** and vimentin **(K)** proteins in mouse lung metastatic sections. Scale bar: 100 μm. **(L-O)** Quantitative analysis of IHC staining for Ki-67 **(L)**, E-cadherin **(M)**, N-cadherin **(N)** and vimentin **(O)** proteins in mouse lung metastatic samples (n = 5 per group). Values are presented as the mean ± SD. Significance was determined using a two-sided t-test in **C** or using one-way ANOVA in **A, B, D, E, F, G, L, M, N** and **O**. *P < 0.05; **P < 0.01; ***P < 0.001; ****P < 0.0001; ns = not significant.

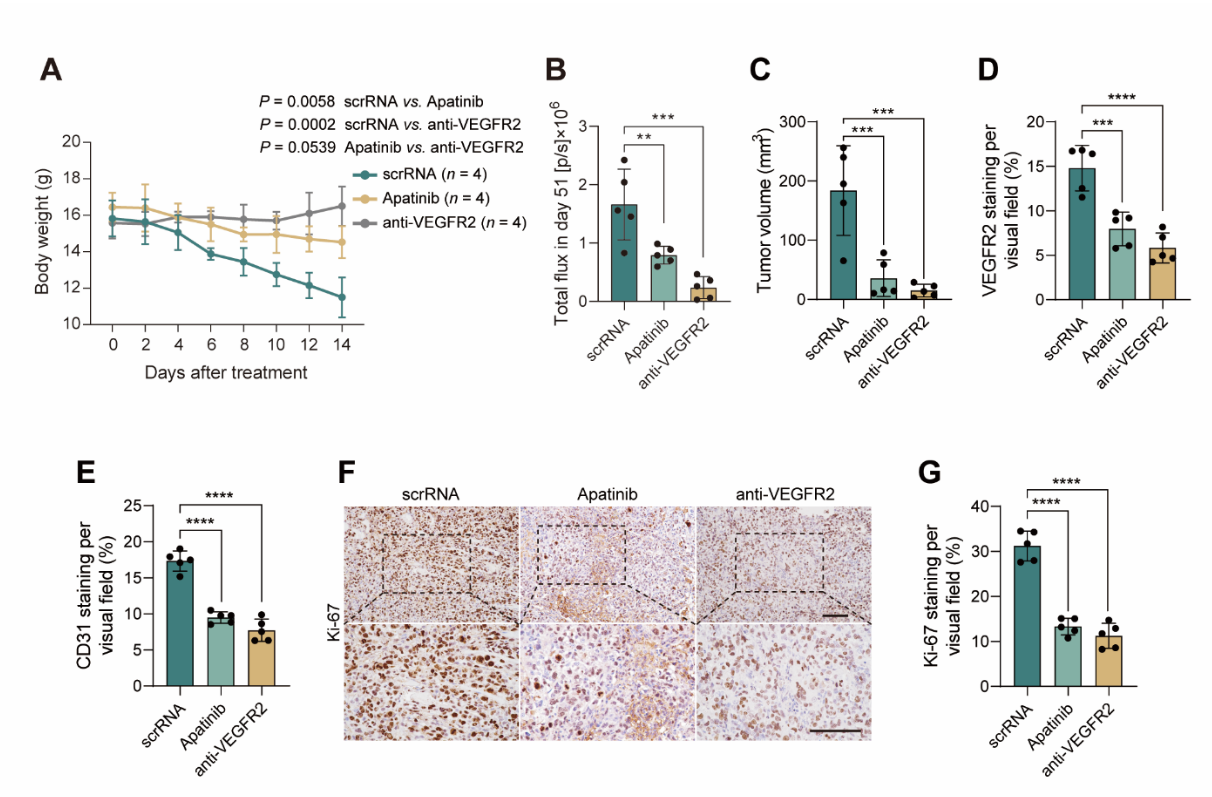

Figure S6. Evaluation of the therapeutic efficacy of self-assembled VEGFR2 siRNA in the spontaneous OS metastasis model.

The OS orthotopic xenograft tumor model was constructed by injecting stable 143B cells labeled with firefly luciferase into the tibial bone marrow cavity of NOD/SCID mice. Thirty days after injection, the tumor-bearing right legs of all mice were amputated aseptically. All mice were randomly divided into two groups for either survival analysis or tumor evaluation according to pulmonary tumor burden. Mice were then intravenously injected with the scrRNA circuit or anti-VEGFR2 circuit (10 mg/kg) or intragastrically administered 200 mg/kg apatinib every 2 days for a total of seven treatments. After treatment, the survival condition, tumor growth and VEGFR2 expression levels were evaluated. **(A)** Body weight curves (n = 4 per group). **(B)** Luminescence intensity quantification of 143B cells in the lung from mice posttreatment (day 51) with the genetic circuits or apatinib (n = 5 per group). **(C)** Semiautomated quantitative image analysis of tumor volumes posttreatment with genetic circuits or apatinib using 3-D reconstructions (n = 5 per group). **(D-E)** Quantitative analysis of IHC staining for VEGFR2 and CD31 proteins in mouse lung metastatic samples (n = 5 per group). **(F)** Representative images of IHC staining for Ki-67 protein in mouse lung metastatic sections. Scale bar: 100 μm. **(G)** Quantitative analysis of IHC staining for Ki-67 protein in mouse lung metastatic samples (n = 5 per group). Values are presented as the mean ± SD. Significance was determined using one-way ANOVA in **A, B, C, D, E** and **G**. ***P < 0.001; ****P < 0.0001.

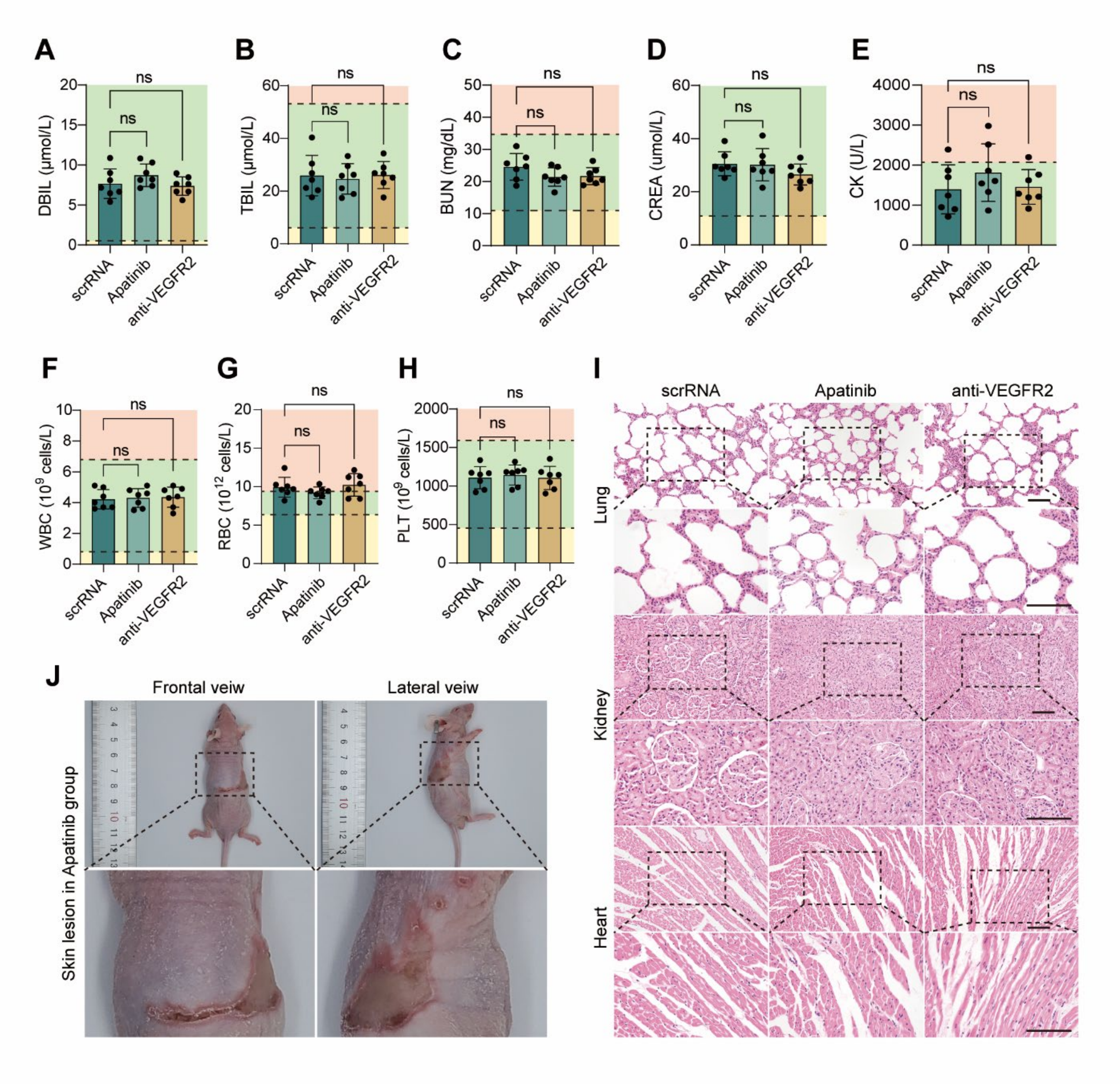

Figure S7. Evaluation of the toxic effects and tissue damage of self-assembled VEGFR2 siRNA *in vivo*.

C57BL/6J mice were intravenously injected with scrRNA circuit or anti-VEGFR2 circuit (10 mg/kg) or intragastrically administered 200 mg/kg apatinib every 2 days for a total of seven times. After treatment, mice were sacrificed, and blood and tissue samples were collected and analyzed for serum biochemical indicators, peripheral blood cell counts and tissue damage. **(A-E)** Biochemical indexes measured in serum include (n = 7 per group): **(A)** DBIL (normal range: 0.45-33.89 μmol/L), **(B)** TBIL (normal range: 6.09-53.06 μmol/L), **(C)** BUN (normal range: 10.81-34.74 mg/dL), **(D)** CREA (normal range: 10.91-85.09 μmol/L) and **(E)** CK (normal range < 2070.55 U/L).

(F-H) Counts of WBCs, RBCs and PLTs measured in peripheral blood (n = 7 per group): **(F)** WBCs (normal range: 0.8-6.8 $\times 10^9$ /L), **(G)** RBCs (normal range: 6.36-9.42 $\times 10^{12}$ /L) and **(H)** PLTs (normal range: 450-1590 $\times 10^9$ /L). Red, green and yellow shaded areas in **A-H** represent the high level, normal level and low level respectively. Two dotted lines represent the lower and upper limits of the normal range.

(I) Histological examination of the lung, kidney and heart from genetic circuits or apatinib-treated mice. No overt tissue damage was observed in any of the tissues. Scale bar: 100 μm. **(J)** Gross specimens of the skin reactivity of genetic circuits or apatinib-treated mice: front view (left) and side view (right). Values are presented as the mean ± SD. Significance was determined using one-way ANOVA in **A, B, C, D**, **E, F, G** and **H**. ns = not significant.

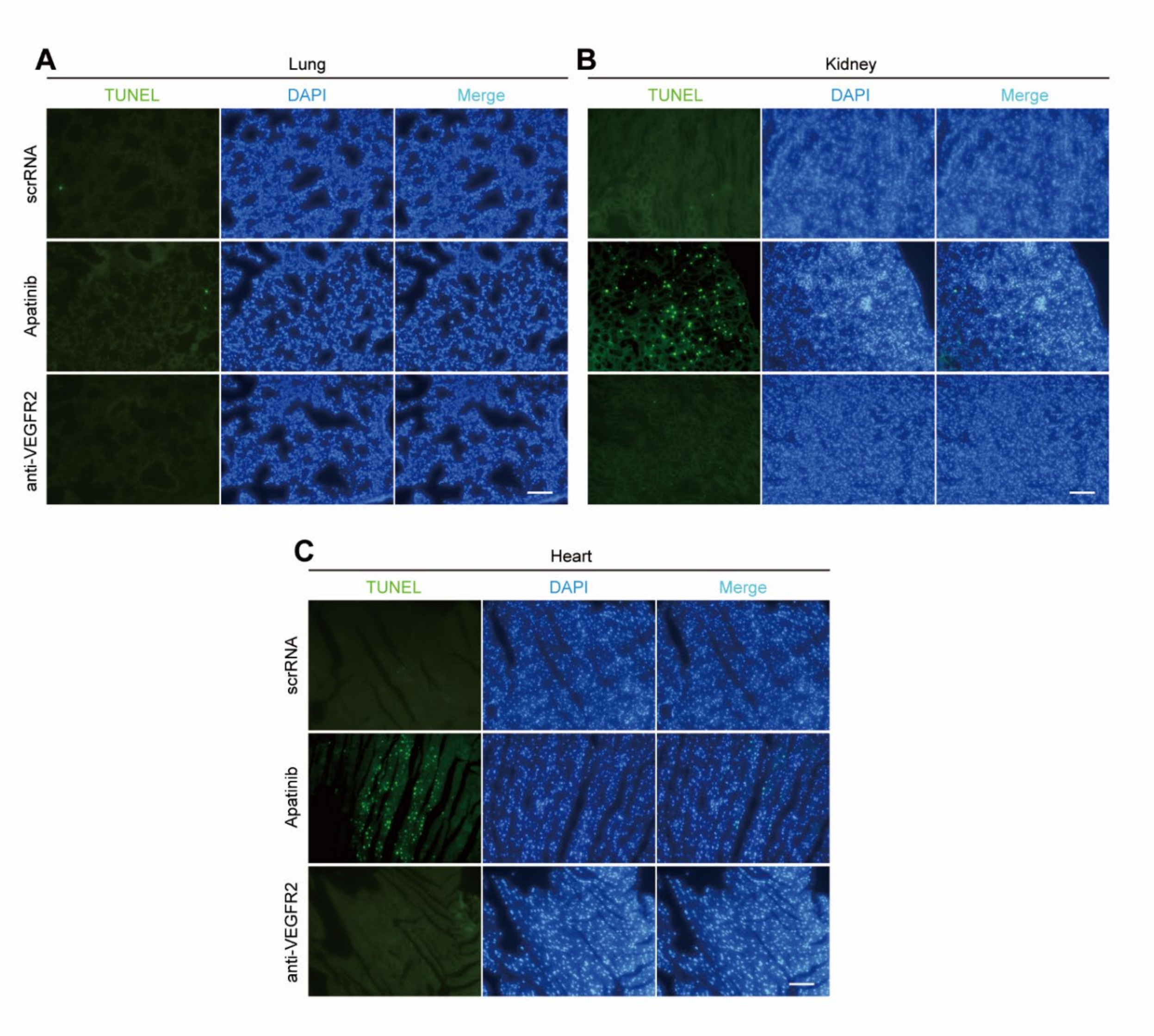

Figure S8. Evaluation of the toxic effects and tissue damage of self-assembled VEGFR2 siRNA *in vivo*.

Representative images of TUNEL stained images of the lungs **(A)**, kidneys **(B)** and hearts **(C)** from genetic circuits or apatinib-treated mice. Scale bar: 100 μm.

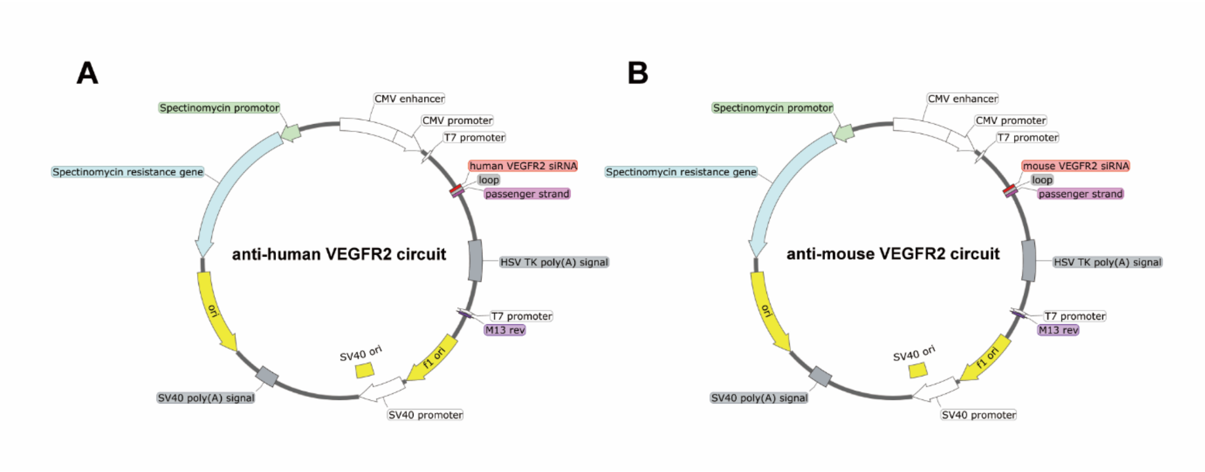

Figure S9. Maps of the plasmids used to express genetic circuits.

Maps of the plasmids used to express the anti-VEGFR2 circuit of human origin **(A)** and mouse origin **(B)**.
