# Supplementary material for: In vivo self-assembly and delivery of VEGFR2 siRNA-encapsulated small extracellular vesicles for lung metastatic osteosarcoma therapy
Source: Cell Death Dis. 2023 Sep 22;14(9):626. doi: 10.1038/s41419-023-06159-3 (PMC10516902; doi:10.1038/s41419-023-06159-3)
Supplement: Supplementary file 2 — Raw data of WB [file 41419_2023_6159_MOESM2_ESM.pdf]

In vivo self-assembly and delivery of VEGFR2 siRNA-encapsulated small extracellular vesicles for lung metastatic osteosarcoma therapy

Raw data of Western blots

Lingfeng Yu†, Gentao Fan†, Qingyan Wang, Yan Zhu, Hao Zhu, Jiang Chang, Zhen Wang, Shoubin Zhan, Xianming Hua, Diankun She, Jianhao Huang, Yicun Wang, Jianning Zhao, Chen-Yu Zhang*, Xi Chen*, Guangxin Zhou*

This PDF file includes:

Full and uncropped western blots of Figures 2D, Figure 2F, Figure 2I, Figure 5G, Figure S1D, Figure S1G, Figure S1H, Figure S2E, Figure S2H

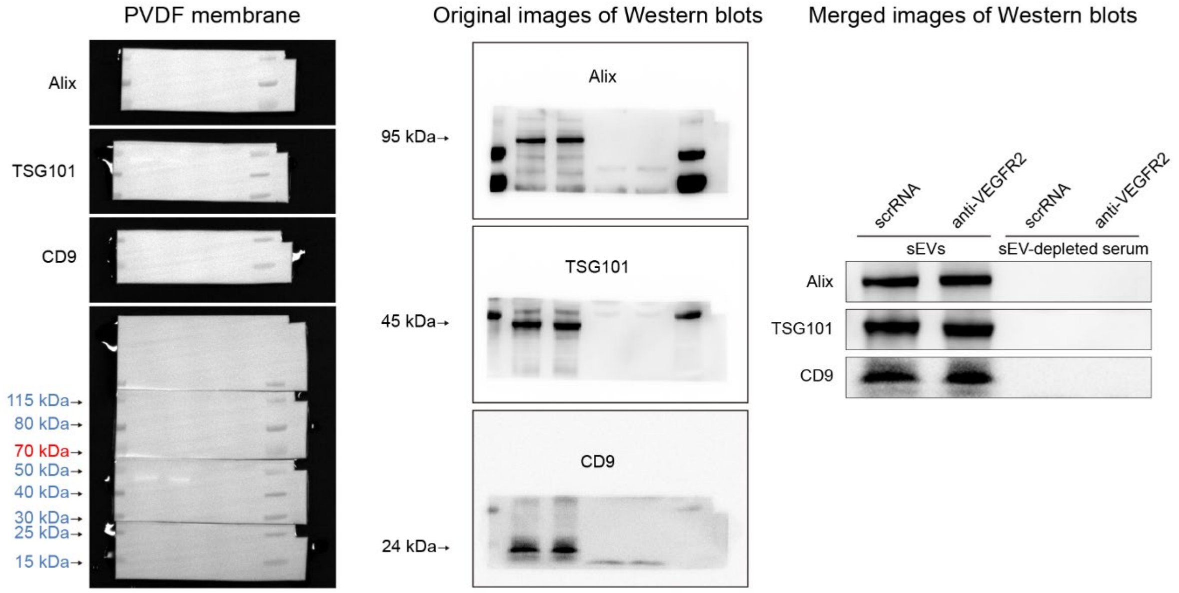

Figure 2D. Western blot analysis of specific sEV markers (Alix, TSG101 and CD9) in purified sEVs and sEV-depleted serum.

After the scrRNA or anti-VEGFR2 circuits treatment, sEVs were purified from mouse serum and subjected to western blot analysis. The PVDF membranes were cut based on the apparent molecular weight of Alix (~95 kDa), TSG101 (~ 45 kDa) and CD9 (~ 24 kDa), and the bands containing proteins of interest were blotted with anti-Alix, anti-TSG101 and anti-CD9 antibodies, respectively, and detected with a fluorescent secondary antibody. sEV-depleted serum served as the negative control. PageRuler Prestained Protein Ladder (26616, Thermo Fisher Scientific) was applied.

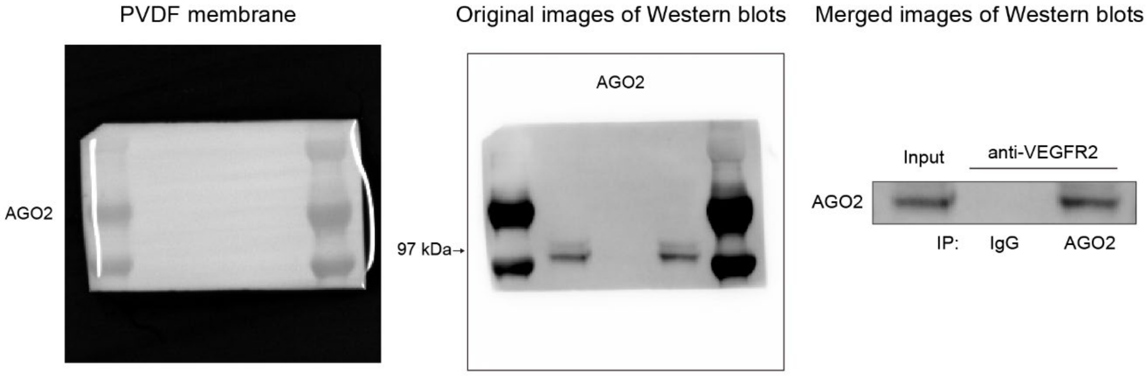

Figure 2F. Western blot analysis of AGO2 protein level in serum sEVs purified from mice injected with anti-VEGFR2 circuit.

The PVDF membrane was cut based on the apparent molecular weight of AGO2 (~97 kDa), and the bands containing proteins of interest were blotted with anti-AGO2 antibody, and detected with a fluorescent secondary antibody. IgG served as a negative control. PageRuler Prestained Protein Ladder (26616, Thermo Fisher Scientific) was applied.

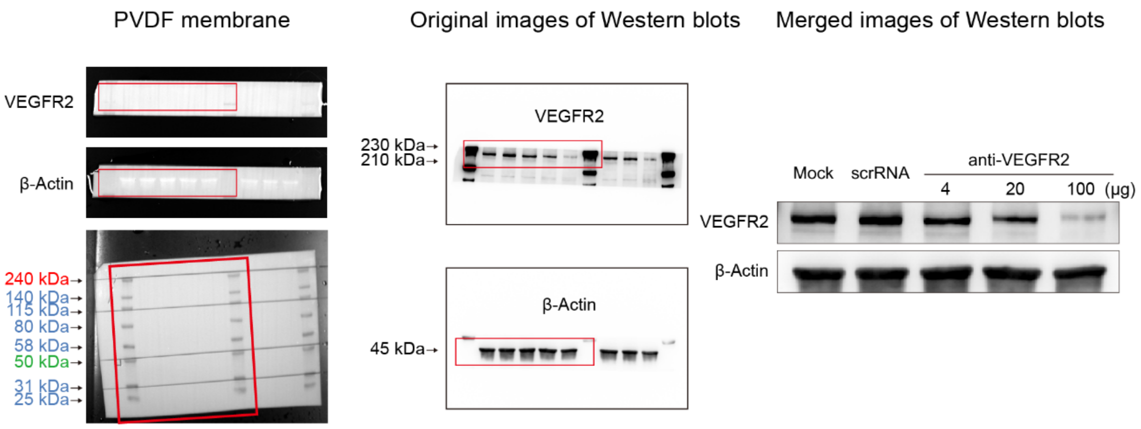

Figure 2I. Western blot analysis of VEGFR2 protein level in HUVEC cells under incubation with serum sEVs purified from mice injected with genetic circuit.

HUVEC cells were subjected to western blot analysis after 36-h incubation with serum sEVs purified from mice injected with scrRNA circuit or anti-VEGFR2 circuit. Different doses of sEVs were added to reveal the dose-dependent effect. The PVDF membranes were cut based on the apparent molecular weight of VEGFR2 (~230/210 kDa) and β-Actin (~ 45 kDa), and the bands containing proteins of interest were blotted with anti-VEGFR2 and anti-β-Actin antibodies, respectively, and detected with a fluorescent secondary antibody. Prestained Color Protein Ladder (6.5-270kDa, Beyotime) was applied. Red frame represents the components of merged images in Figure 2I.

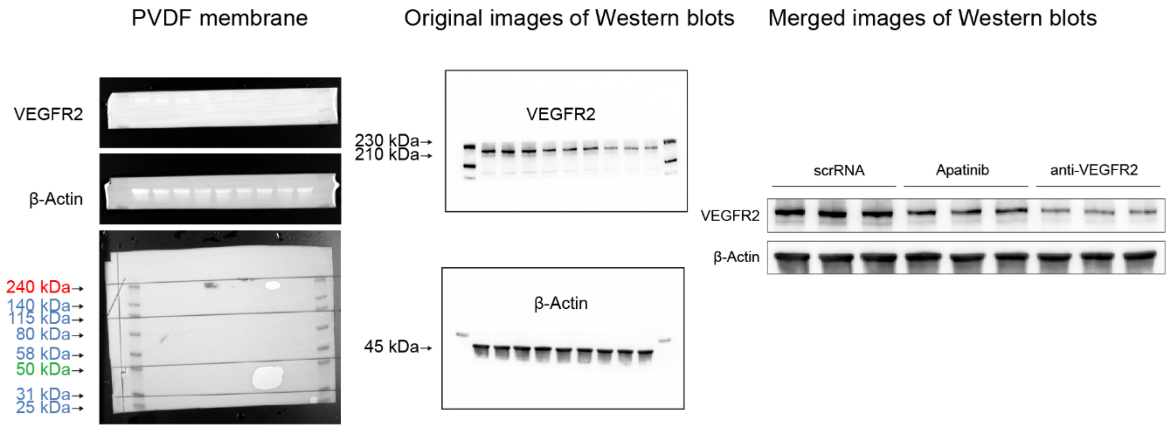

Figure 5G. Western blot analysis of VEGFR2 protein level in lung metastatic samples from the OS lung metastasis model.

Mice were intravenously injected with the scrRNA circuit or anti-VEGFR2 circuit (10 mg/kg) or intragastrically administered with 200 mg/kg Apatinib every 2 days for a total of 7 treatments, and then mice were sacrificed and the lung metastatic lesions were collected for western blot analysis. The PVDF membranes were cut based on the apparent molecular weight of VEGFR2 (~230/210 kDa) and β-Actin (~ 45 kDa), and the bands containing proteins of interest were blotted with anti-VEGFR2 and anti-β-Actin antibodies, respectively, and detected with a fluorescent secondary antibody. Prestained Color Protein Ladder (6.5-270kDa, Beyotime) was applied.

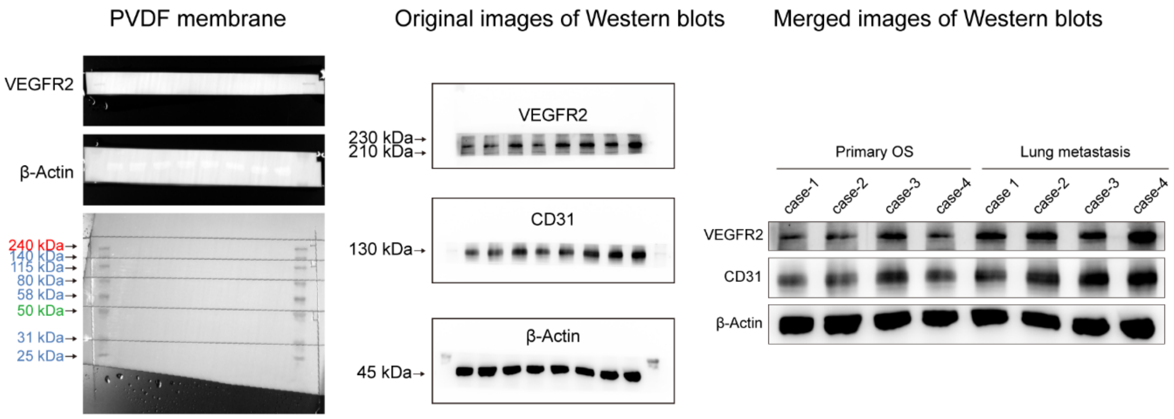

The PVDF membranes were cut based on the apparent molecular weight of VEGFR2 (~230/210 kDa), CD31 (~ 130 kDa) and β-Actin (~ 45 kDa), and the bands containing proteins of interest were blotted with anti-VEGFR2, anti-CD31 and anti-β-Actin antibodies, respectively, and detected with a fluorescent secondary antibody. Prestained Color Protein Ladder (6.5-270kDa, Beyotime) was applied. PVDF membrane of CD31 was not photographed due to negligence.

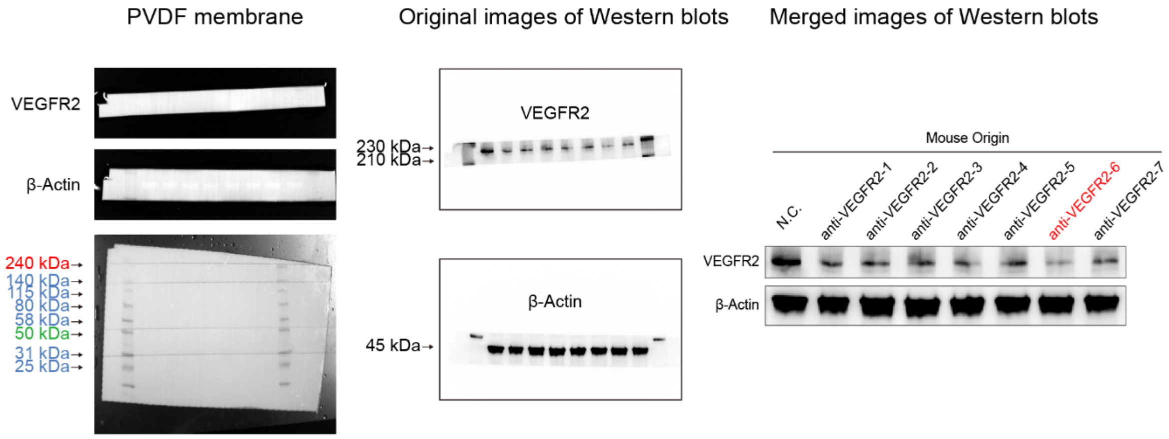

Figure S1G. Western blot analysis of the knockdown effect of candidate VEGFR2 siRNAs of mice origin in anti-VEGFR2 circuits.

The candidate circuit coding mouse VEGFR2 siRNA was transfected into murine endothelial cells (EOMA) for western blot analysis. The PVDF membranes were cut based on the apparent molecular weight of VEGFR2 (~230/210 kDa) and β-Actin (~ 45 kDa), and the bands containing proteins of interest were blotted with anti-VEGFR2 and anti-β-Actin antibodies, respectively, and detected with a fluorescent secondary antibody. Prestained Color Protein Ladder (6.5-270kDa, Beyotime) was applied.

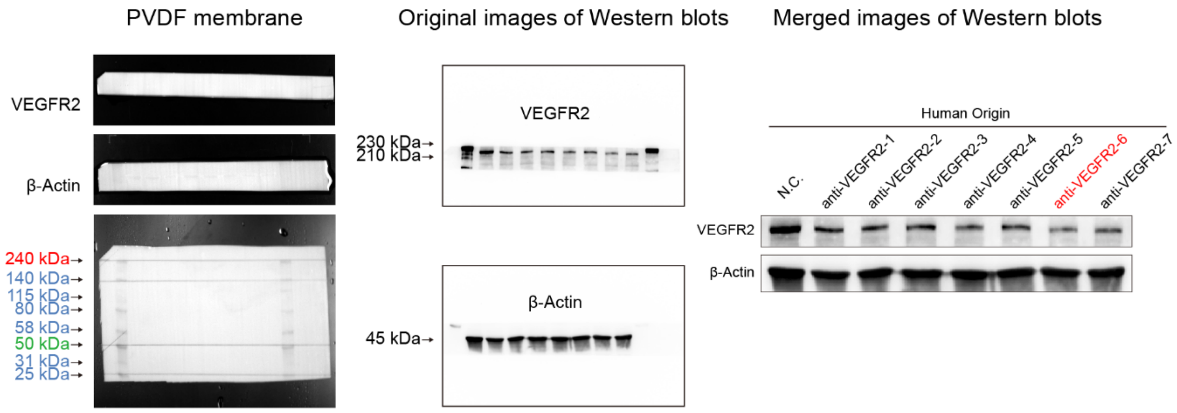

Figure S1H. Western blot analysis of the knockdown effect of candidate VEGFR2 siRNAs of human origin in anti-VEGFR2 circuits.

The candidate genetic circuit coding human VEGFR2 siRNA was transfected into HUVEC cells for western blot analysis. The PVDF membranes were cut based on the apparent molecular weight of VEGFR2 (~230/210 kDa) and β-Actin (~ 45 kDa), and the bands containing proteins of interest were blotted with anti-VEGFR2 and anti-β-Actin antibodies, respectively, and detected with a fluorescent secondary antibody. Prestained Color Protein Ladder (6.5-270kDa, Beyotime) was applied.

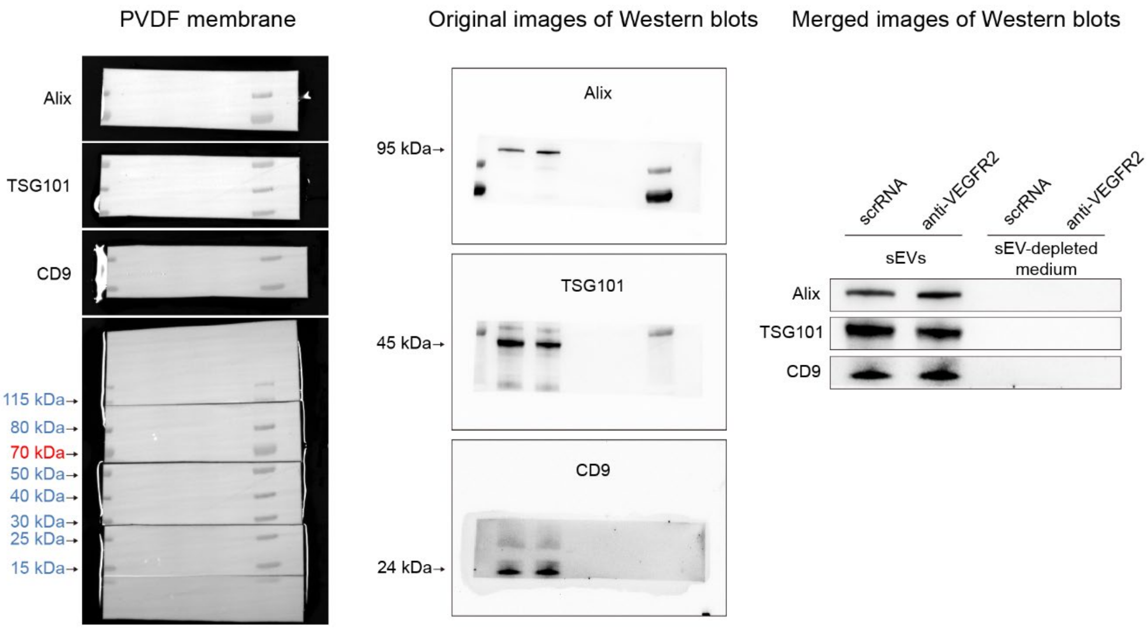

Figure S2E. Western blot analysis of specific markers (Alix, TSG101 and CD9) in hepatocytes-secreted sEVs. C57BL/6J mice were intravenously injected with scrRNA circuit or anti-VEGFR2 circuit (10 mg/kg) every 2 days for a total of seven times, and then the primary hepatocytes were extracted from the mouse livers. The sEVs were purified from the primary hepatocyte culture supernatant for western bolt analysis. The PVDF membranes were cut based on the apparent molecular weight of Alix (~95 kDa), TSG101 (~ 45 kDa) and CD9 (~ 24 kDa), and the bands containing proteins of interest were blotted with anti-Alix, anti-TSG101 and anti-CD9 antibodies, respectively, and detected with a fluorescent secondary antibody. sEV-depleted culture supernatant served as the negative control. PageRuler Prestained Protein Ladder (26616, Thermo Fisher Scientific) was applied.

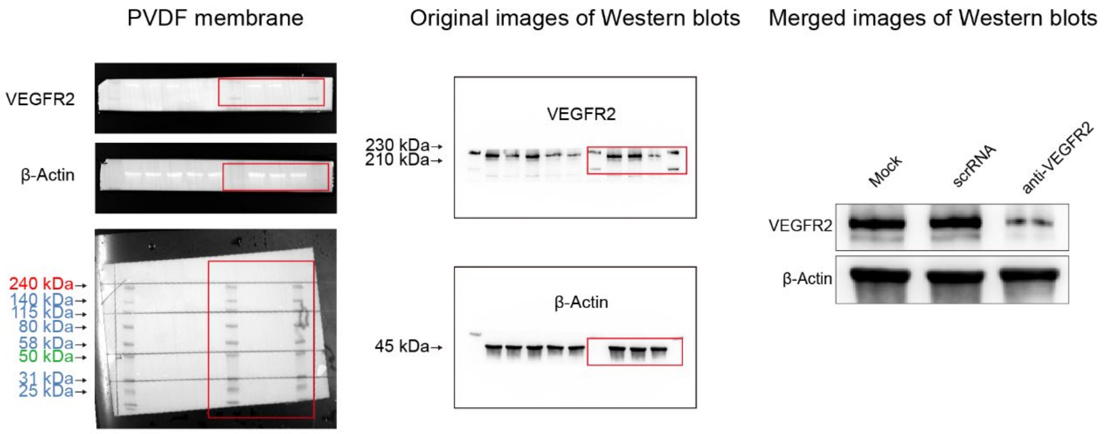

Figure S2H. Western blot analysis of VEGFR2 protein levels in HUVEC cells after 36-h incubation with hepatocytes-secreted sEVs.

C57BL/6J mice were intravenously injected with scrRNA circuit or anti-VEGFR2 circuit (10 mg/kg) every 2 days for a total of seven times, and then the primary hepatocytes were extracted from the mouse livers. The sEVs purified from the primary hepatocyte culture supernatant were incubated with HUVEC cells for western blot analysis. The PVDF membranes were cut based on the apparent molecular weight of VEGFR2 (~230/210 kDa) and β-Actin (~ 45 kDa), and the bands containing proteins of interest were blotted with anti-VEGFR2 and anti-β-Actin antibodies, respectively, and detected with a fluorescent secondary antibody. Prestained Color Protein Ladder (6.5-270kDa, Beyotime) was applied. Red frame represents the components of merged images in Figure S2H.
